# Experimental Investigation into the Design, Optimization, Toxicity, and Anti-Viral Efficacy of Proliposomes Loaded with Ivermectin Against Infectious Bronchitis Virus Using an Embryonated Chicken Egg Model

**DOI:** 10.3390/pharmaceutics17020165

**Published:** 2025-01-25

**Authors:** Mohammad H. Alyami, Hamad S. Alyami, Asmaa M. Abdo, Shereen A. Sabry, Shimaa M. G. Mansour, Hanan M. El-Nahas, Margrit M. Ayoub

**Affiliations:** 1 Department of Pharmaceutics, College of Pharmacy, Najran University, Najran 66462, Saudi Arabia; 2 Department of Pharmaceutics, Faculty of Pharmacy, Zagazig University, Zagazig 44519, Egypt; 3 Department of Virology, Faculty of Veterinary Medicine, Zagazig University, Zagazig 44511, Egypt

**Keywords:** infectious bronchitis virus, proliposomes, ivermectin, mannitol, stearyl glycyrrhetinate

## Abstract

**Background**: Infectious bronchitis virus (IBV) causes a significant illness in birds, making it a leading source of financial loss in the poultry business. The objective of this study was to assess the effectiveness of proliposomes (PLs) containing ivermectin (IVM) against IBV using embryonated chicken eggs (ECEs). **Methods**: A three-factor, two-level (2^3^) full factorial design was employed; carrier/lipid phase ratio (A), stearyl glycyrrhetinate amount (B), and phospholipid type (C) were studied as independent variables, while product yield (PY), entrapment efficiency (EE), particle size (PS), polydispersity index (PDI), zeta potential (ZP), and cumulative percentage of drug released after 6 h (Q6h) were characterized. The selected formulations (PL2 and PL6) were subjected to further characterizations, including IVM toxicity and anti-viral activity. **Results**: The PY% ranged from 88.6 ± 2.19% to 98.8 ± 0.45%, EE% was from 71.8 ± 2.01% to 96.1 ± 0.51%, PS was from 330.1 ± 55.65 nm to 1801.6 ± 45.61 nm, PDI was from 0.205 ± 0.06 to 0.603 ± 0.03, ZP was from −18.2 ± 0.60 mV to −50.1 ± 1.80 mV, and Q6h was from 80.95 ± 1.36% to 88.79 ± 2.03%. IVM-loaded PLs had lower toxicity in ECEs than pure IVM; the mortality rate was substantially reduced in IBV-infected ECEs injected with PL2 rather than pure IVM. As further evidence of IVM’s anti-viral action against IBV, quantitative real-time polymerase chain reaction (qRT-PCR) revealed that the PL2-treated group exhibited further reduction in IBV’s copies in comparison with the pure IVM-treated group. **Conclusions**: PLs loaded with IVM are an innovative and potentially effective way to inhibit IBV.

## 1. Introduction

The poultry industry is being threatened financially by avian infectious bronchitis virus (IBV), which is the coronavirus of the chicken (*Gallus gallus*), and causes an acute, highly contagious upper respiratory tract disease. IBV affects the performance of both meat-type and egg-laying birds and chickens of all ages [1]. No risks to human health are suspected or have been demonstrated to arise from IBV [2]. IBV and other avian coronaviruses of turkeys and pheasants are classified as *Gammacoronaviruses*, while mammalian coronaviruses comprise *Alpha* and *Betacoronaviruses* [3].

The COVID-19 pandemic spread across the world at an alarming rate in the first quarter of 2020. The research for treating the condition sparked intense attention in the medical world. There have been many attempts at repurposing existing and approved drugs for the treatment of COVID-19. Anti-virals, anti-parasitics, and anti-inflammatory drugs were among those that attracted a lot of media attention. Remdesivir, baricitinib, choloroquine/hydroxychloroquine, famotidine, ivermectin, and other drugs were tested [4].

Ivermectin (IVM) is widely used in both animals and humans as a Food and Drug Administration (FDA)-approved parasiticide [5]. It is the common name of 22,23-dihydroavermectins B1 and it is a semi-synthetic derivative of avermectins that is produced from naturally occurring fermentation products [6]. Ivermectin is a broad spectrum anti-parasitic agent belonging to the macrocyclic lactone group; it treats several neglected tropical diseases, including onchocerciasis, helminthiases, scabies, and other parasitic infections [7]. It is widely used in low- and middle-income countries (LMICs) to treat worm infections [8]. IVM is a non-hygroscopic crystalline powder that belongs to class II/IV in the biopharmaceutics classification system (BCS) [9]. It has limited aqueous solubility; consequently, it has low oral bioavailability [10].

There are several studies focusing on the anti-viral activity of IVM with demonstrated pre-clinical activity against chikungunya virus [11], pseudorabies virus [12], West Nile virus [13], and more lately, in vitro activity against SARS-CoV-2, the causative agent of COVID-19 [14]. Until now, the FDA has not approved or authorized IVM for use in preventing or treating COVID-19 in humans or animals; the evidence on IVM for the prevention of severe acute respiratory syndrome (SARS)-CoV-2 infection and COVID-19 treatment is still conflicting [5]. The National Institutes of Health in the United States recently stated that “There are insufficient data to recommend either for or against the use of IVM for the treatment of COVID-19”; the world health organization (WHO) recommends against its use outside of clinical trials [15].

Although there is demonstrated efficacy of IVM, its activity was limited because of some critical issues related to the currently available dosage forms, i.e., tablets, capsules, and suspensions [16]. Therefore, several studies were conducted to formulate IVM in micro- and nanoparticles including polymer nanocapsules [17], chitosan–alginate nanoparticles [18], nanolipid carriers [19], and self-implanted tiny needles [20].

There are many studies on the role of licorice extract in managing COVID-19 [21]. The main triterpenoid active constituents of licorice extract are glycyrrhizin (glycyrrhizic acid), glycyrrhetinic acid, and its derivatives [22]. It was shown that glycyrrhizic acid could inhibit coronavirus replication in vitro [23]. It was reported that using a small dose of glycyrrhizin, about 10–50 mg, in licorice extract as a daily prophylactic dose and a large dose of about 50–100 mg three times a day can prevent the progression of the disease during its initial phase [22]. Glycyrrhizin is a conjugate of glycyrrhetinic acid and two molecules of glucuronic acid. Glycyrrhetinic acid is a pentacyclic lactone with a structure similar to that of cortisone, so it is responsible for the anti-inflammatory effect of licorice extract [24]. Stearyl glycyrrhetinate (SG) is the salt and ester of glycyrrhetinic acid.

Liposomes are a lipid-based drug delivery system; they were found to be the most promising drug delivery system to encapsulate the lipophilic drugs [25]. Phospholipid degradation by hydrolysis or oxidation in aqueous dispersion followed by aggregation, sedimentation, and leakage of the encapsulated drug is the main drawback of liposomes [26]. Proliposomes (PLs) are a novel type of carrier-mediated drug delivery system; they are a promising strategy to improve liposomes’ physicochemical properties and stability [27]. Proliposomes are dry, free-flowing particles that immediately form liposomal dispersion of multilamellar vesicles upon contact with water or biological fluid; the resulting liposomal vesicles are similar to conventional liposomes and more uniform in size [28,29]. The superiority of PLs over liposomes for drug delivery is due to their stability [29]. Moreover, the solubility and bioavailability problems of many drugs can be overcome by developing PLs formulations [30].

No research has been published on enhancing the action of IVM through its formulation in a PLs system. This study set out to formulate, optimize IVM-loaded PLs and SG-modified IVM-loaded PLs, and investigate their anti-viral efficacy against avian IBV using ECEs. For this purpose, different variables influencing vesicles’ characteristics were studied employing 2^3^ full factorial design using Design Expert^®^ software, version 11 to identify the optimized formulation. The selected formulations were subjected to further characterizations, such as scanning electron microscopy (SEM), differential scanning calorimetry (DSC), Fourier transform infrared spectroscopy (FTIR), and stability studies; additionally, investigations into IVM toxicity and anti-viral activity against IBV-infected ECEs were performed.

## 2. Materials and Methods

### 2.1. Materials

Ivermectin was kindly supplied by Delta Pharma Co., Cairo, Egypt. Soybean phosphatidylcholine (SPC; Lipoid S-100) was kindly gifted by Lipoid, Steinhausen, Switzerland. Dipalmitoyl phosphatidylcholine (DPPC) was obtained from Avanti Polar Lipids (Alabaster, AL, USA). Stearyl glycyrrhetinate was supplied by Tokyo Chemical Industry Co., Ltd., Tokyo, Japan. Cholesterol was purchased from BDH Chemicals Ltd., London, UK. Ethanol, potassium di-hydrogen orthophosphate, di-sodium hydrogen orthophosphate, and sodium lauryl sulfate (SLS) were purchased from El-Naser pharmaceuticals Chemicals Co. (Cairo, Egypt). All other chemicals were of analytical grade and used as received.

### 2.2. Methods

#### 2.2.1. Experimental Design

The IVM-loaded PLs were prepared following the three-factor, two-level (2^3^) full factorial experimental design using Design-Expert^®^ software (version 11, Stat-Ease Inc., Minneapolis, MN, USA). The experimental design is a scientific technique used to study the effect of the independent variables (factors) on the measured dependent variables (responses). Carrier/lipid phase ratio (A), stearyl glycyrrhetinate amount (B), and phospholipid type (C) were selected as independent variables. In addition, the measured responses were set, including PY% (Y_1_), EE% (Y_2_), PS (Y_3_), PDI (Y_4_), ZP (Y_5_), and Q6h (Y_6_). The goal was to optimize a PLs formulation loaded with IVM to have favorable characteristics regarding the goal criteria that could achieve the study aim. The two different levels of the independent factors and the goal criteria for the measured responses are shown in Table 1. An analysis of variance (ANOVA) test was adopted, measuring several parameters such as the multiple correlation coefficient (R^2^), adjusted R^2^, predicted R^2^, and adequate precision, to analyze the obtained data for assessing the model significance and to prove the statistical analysis of the data. Moreover, the graphs of 3-D response surface plots and one-factor plots were studied to assess the significant relationship between the studied factors and the measured responses, and the linearity plots of the observed versus predicted values were studied to ensure the validity of the chosen model for different responses.

#### 2.2.2. Preparation of IVM-Loaded PLs Formulations

The IVM-loaded PLs formulations were prepared using various ratios of SPC, DPPC, cholesterol, mannitol, and SG (Table 2). The lipid phase (300 mg) consisted of 210 mg SPC or DPPC and 90 mg of cholesterol. The PLs were prepared with a range of carrier/lipid phase ratio (6:1 and 9:1 *w*/*w*) via the slurry method, as reported previously by Khan et al. [31] with minor modifications. This was achieved by coating 300 mg of lipid phase onto 1800 mg or 2700 mg of mannitol. Firstly, the carbohydrate carrier (mannitol) was sieved with 100 mesh and placed in a 50 mL pear-shaped flask, which was held at 70 °C, and the flask was rotated at 90 rpm using the rotary evaporator (Basis Hei-VAP HL, Heidolph Instruments GmbH & Co. KG, Schwabach, Germany). The rotating flask was kept in a water bath (Heizbad Hei-VAP, Heidolph Instruments GmbH & Co. KG, Schwabach, Germany) and mannitol was dried under vacuum for 30 min. Briefly, the required amounts of IVM (6 mg), phospholipid, cholesterol, and SG were weighed and dissolved in ethanol (333 mg/mL). The resultant mixture was transferred to the pear-shaped flask after mannitol drying. A rotary evaporator was used to evaporate the ethanol from the slurry, using a vacuum pump (Buchi Vac V-501, Buchi AG, Flawil, Switzerland), and to set up the water bath of the evaporator at 45 °C and the rotation speed at 250 rpm for 2 h. The PLs granules were harvested and sieved with 100 mesh, then collected and stored at −18 °C for conducting the subsequent studies.

#### 2.2.3. Characterization of IVM-Loaded PLs Formulations

##### Estimation of Product Yield (PY%)

After preparation of the IVM-loaded PLs, it was collected and weighed accurately. The product yield was determined according to the following equation [28]:

PY (%) = (W°/WT) × 100

where W° = total weight of PLs, WT = sum of initial weight of mannitol, phospholipid, cholesterol, and drug(s) employed.

##### Estimation of Entrapment Efficiency (EE%)

After hydrating 100 mg of PLs with 10 mL of distilled water (DW) [8], vortex-mixing was conducted for two minutes to ensure complete dissolution of the carrier particles and hydration/dispersion of the lipid [31]. The EE of IVM in the reconstituted liposomes was assessed via centrifugation of the liposomal suspension for 60 min at 4 °C and 13,000 rpm. In order to remove the unentrapped (free) drug, the clear supernatant was siphoned off and the pellets were resuspended and centrifugated again to ensure that the free drug was completely removed. At λmax (246 nm), the free drug concentration was determined spectrophotometrically [25].

The EE% was calculated according to the following equation [32]:

EE (%) = [(total drug loading − unentrapped drug)/total drug loading] × 100

##### Estimation of Particle Size (PS) and Polydispersity Index (PDI)

The PS and PDI of liposomes generated upon hydration of the prepared PLs were analyzed by laser diffraction according to the method reported by Byeon et al. [33]. Proliposomes (20 mg) were mixed with 10 mL of DW and dispersed well to obtain homogenous particles; then, the PS was measured by a computerized Malvern Zetasizer Nano-ZS90 (Malvern Instruments Ltd., Malvern, UK). The measurements were carried out in triplicate after equilibration at 25 °C for 2 min.

##### Zeta Potential Measurement (ZP)

The ZP of vesicles was determined using a computerized Malvern Zetasizer Nano-ZS90 (Malvern Instruments Ltd., Malvern, UK) by selecting the instrument’s relevant software option. Liposomes generated upon hydration of the prepared PLs were loaded into the Malvern’s zeta potential cells. ZP was determined after setting the temperature at 25 °C and allowing 2 min for sample equilibration, in order to obtain consistent ZP.

##### Cumulative Percentage of Drug Released After 6 h (Q6h)

The release of IVM from the prepared formulations was performed using the dialysis bag method according to the method conducted by Karn et al. [34] with minor modifications. The PLs formulations equivalent to 4 mg of drug were sealed in dialysis bags by tightening the two ends of the tube with a thin thread. Then, each dialysis bag containing a sample was immersed in a glass bottle containing 20 mL of phosphate buffer pH 6.8 containing 0.5% (*w*/*v*) of sodium lauryl sulfate. The bottles were shaken at 50 rpm and 37 ± 0.5 °C. Aliquots of 1 mL were taken from each bottle at different time intervals (0.5, 1, 1.5, 2, 3, 4, 5, and 6 h). Samples were analyzed for the drug content at 246 nm using phosphate buffer pH 6.8 containing 0.5% SLS as a blank. The dissolution medium was replaced with fresh medium to maintain the sink condition. The process of buffer preparation, dissolution studies, and analytical procedures were all conducted at 37 ± 0.5 °C. Each sample was run in triplicate. Then, the standard error of mean values was calculated.

##### Kinetic Study of Drug Release

To determine the in vitro release mechanism of all PLs formulations, the release data were subjected to the following models: zero order model (Q_t_ = K_o_·t), first order model (log Q_t_ = log Q_o_ − K·t/2.303), Higuchi release model (Q_t_ = K_H_·t0.5), Korsmeyer–Peppas model (Q_t_/Q_∞_ = K_kp_·tn), and Hixson–Crowell model (Qo^1/3^ − Qt^1/3^ = K_s_·t) where Q_t_ is the amount of drug released at time (t), Q_o_ is the initial drug amount, Q_∞_ is the amount of drug released at time infinity (∞), K_o_, K, k_H_, K_kp_, K_s_ are the release rate constants of the previous models, respectively, and n is the release exponent. The model of highest correlation coefficient (R^2^) was considered as the best fit model. Furthermore, the mechanism of drug release was confirmed by the n value of the Korsmeyer–Peppas model [35].

#### 2.2.4. Optimization Technique

The optimization process was performed after the statistical analysis. The optimized formulation was selected by the design software depending on the goal criteria, which is the minimization of PS and PDI and maximization of PY%, EE%, ZP (as an absolute value), and Q6h, as investigated in Table 1. The validity of the models was determined according to the optimized formulation’s desirability value and the closeness of experimental values to the predicted ones by the design [36]. The optimized formulation was evaluated by further characterization tests.

#### 2.2.5. Characterization of the Selected Formulations

##### Scanning Electron Microscopy (SEM)

The surface morphology of the selected formulations (PL2 and PL6) and pure mannitol was examined using SEM. The samples were sprinkled onto a cupper stub and coated with gold by a sputter coater under a high vacuum. The particles were observed and images were captured under SEM (JEOL JSM 6510 Iv; JEOL, Tokyo, Japan) at an accelerating voltage of 20–30 KV.

##### Differential Scanning Calorimetry (DSC)

Differential scanning calorimetric analysis was conducted to investigate the solid-state properties of the selected formulations. The DSC thermograms of pure IVM, SG, SPC, cholesterol, mannitol, the selected formulations (PL2 and PL6), and the blank formulation were carried out using a DSC instrument (DSC 60, Shimadzu Co., Kyoto, Japan). About 2 mg of each sample was placed in an aluminum pan and sealed. Each sample was heated to 200 °C at a ramping speed of 10 °C/min under a nitrogen purge (20 mL/min).

##### Fourier Transform Infrared Spectroscopy (FTIR)

In order to determine drug–excipient compatibility, comparisons were made between the FTIR spectra of pure IVM, SG, SPC, cholesterol, mannitol, the selected formulations (PL2 and PL6), and the blank formulation by using a Perkin-Elmer FTIR spectrophotometer (series 1600, Perkin-Elmer Corporation, Waltham, MA, USA) under vacuum from 4000 to 400 cm^−1^ with a resolution of 4 cm^−1^ through the potassium bromide disk method.

##### Stability Studies

Stability studies were performed by analyzing the physical appearance, EE, PS, and ZP of selected formulations (PL2 and PL6) upon storage at room temperature 25 ± 1 °C and refrigerated conditions 4 ± 1 °C for 3 months. Samples were examined at 1, 2, and 3 months and the results were compared with the initial measurements of the freshly prepared formulation [37]. Statistical analysis was performed by applying the two-way ANOVA test using GraphPad Prism, version 8.

#### 2.2.6. Investigation of the Anti-Viral Activity Against IBV

##### Ethical Statement

An embryonated chicken eggs (ECEs) model was used to investigate the activity of pure IVM and selected formulations (PL2 and PL6) against IBV (IB MA5). The protocol was approved by the Institutional Animal Care and Use Committee (IACUC) guidelines of the Faculty of Pharmacy, Zagazig University (Approval number: ZU-IACUC/3/F/438/2022).

##### Virus

The Massachusetts type of avian infectious bronchitis live vaccine virus (MSD) was used. The lyophilized vaccine was first reconstituted with 10 mL sterile phosphate-buffered saline (PBS) and then further diluted in PBS to contain 103 EID50 per 100 µL (median egg infective dose 50 per 100 µL).

##### Toxicity Study

Nine-day-old chicken embryos (*Gallus gallus domesticus*) were supplied by a commercial-certified hatchery. Prior to incubation, the eggs were cleaned with 70% ethanol-wetted cotton pieces. Eggs were then candled in a dark room to mark the boundary of the air sac and the head spot using a lead pencil. The maximum non-toxic concentration (MNTC) was determined according to the method previously described by Ghoke et al. [38] with minor modifications. To determine the MNTC of pure IVM, PL2, and PL6, ten-day-old ECEs were divided into four groups (group I, inoculated with pure IVM; group II, inoculated with PL2; group III, inoculated with PL6; and group IV, negative control group).

Serial two-fold dilution of pure IVM, PL2, and PL6 was prepared to achieve 400 µM, 200 µM, 100 µM, 50 µM, 25 µM, 12.5 µM, and 6.25 µM of IVM. Precisely 0.1 mL of two-fold dilution of pure IVM, PL2, and PL6 was inoculated into the allantoic cavity of the ECEs (three eggs for each dilution). The 4th group contained 6 eggs and was kept as a negative control (not receiving any treatment). All the ECEs were placed in an incubator already adjusted at 37 ± 1 °C with a relative humidity of 60–70%.

All the ECEs were candled after 24 h, and any dead embryos were removed. The candling of eggs was conducted twice daily till hatching to check the viability of embryos for different concentrations of pure IVM, PL2, and PL6; the dead embryos were placed in the refrigerator. After 7 days of incubation, two eggs from each dilution of pure IVM, PL2, and PL6 were randomly selected and placed in the refrigerator for chilling at 4 ± 1 °C overnight. Each egg was examined for any sign of lesions.

##### Assay of Anti-Viral Activity

Investigation of the anti-viral activity of pure IVM, PL2, and PL6 against IBV using ECEs was performed according to the method previously described by Azeem et al. [39] with minor modifications. ECEs were prepared as described in the previous section and then divided into five groups (group I, IBV infected and treated with pure IVM; group II, IBV infected and treated with PL2; group III, IBV infected and treated PL6; group IV, IBV infected and not-treated; group V, not-infected and not-treated).

Viral inoculums were injected through the allantoic route in ECEs of group 1 up to 3 and incubated at 37 ± 1 °C. A serial two-fold dilution of pure IVM, PL2, and PL6 was prepared to achieve 200 µM, 100 µM, and 50 µM of IVM. Precisely 0.1 mL of the two-fold dilution of pure IVM, PL2, and PL6 was inoculated into the allantoic cavity of the IBV-infected ECEs (three eggs for each dilution). The 4th group contained 6 eggs and was kept as a positive control (infected, non-treated; injected with 0.1 mL of viral suspension under similar experimental conditions), while the 5th group contained 6 eggs and was kept as a negative control (non-infected, non-treated).

The inoculated ECEs were placed in an incubator already adjusted at 37 ± 1 °C with relative humidity of 60–70% for the period of 5 days. All the ECEs were candled after 24 h, and any dead embryos were removed. The candling of eggs was conducted twice daily to check the viability of embryos, and the dead embryos were placed in the refrigerator. Then, after 5 days of incubation, the live eggs were placed in the refrigerator for chilling at 4 ± 1 °C overnight to examine any viral lesion in the embryos. This assay was performed in two separate trials and the results were statistically analyzed using GraphPad Prism version 8. Statistical significance was assessed by two-way analysis of variance (ANOVA) with Tukey post-hoc test. Values are expressed as mean ± standard error of mean.

##### RNA Extraction, Reverse Transcription, and qRT-PCR

The allantoic fluids were collected 72 h post-inoculation and submitted for quantitative real-time polymerase chain reaction (qRT-PCR) analysis to determine the viral load. Total RNA rapid extraction kits (Qiagen, QIAamp^®^ MinElute^®^ Virus Kits, Hilden, Germany, Cat. # 57704) were used for total RNA extraction from 200 µL of allantoic fluids (according to manufacturer’s instructions).

One-step RT-PCR was conducted according to the manufacturer’s protocol (Reliance One-Step Multiplex Supermax, Bio-Rad, USA, Cat. # 12010176) using a qRT-PCR thermal-cycler system (CFX Opus 96, New York, NY, USA). A forward primer (5′-GCT TTT GAG CCT AGC GTT-3′), a reverse primer (5′-GCC ATG TTG TCA CTG TCT ATT G-3′), and a Taqman^®^ labeled probe (5′-CAC CAC CAG AAC CTG TCA CCT C-BHQ1-3′) were used [40]. Firstly, cDNA was formed using reverse transcriptase at 50 °C for 15 min followed by denaturation at 95 °C for 10 min. Then, 40 cycles of qRT-PCR were conducted by repeating the following steps for each cycle: denaturation at 95 °C for 15 s, annealing at 57 °C for 20 s, and extension at 72 °C for 30 s. For each reaction, the cycle threshold (CT) number was determined [40] and used to calculate amount of cDNA in each sample.

## 3. Results and Discussion

### 3.1. Preliminary Studies for Preparation of IVM-Loaded PLs

Ivermectin-loaded PLs were prepared using the slurry method technique. This method generates liposomal vesicles with smaller size and a higher percentage of drug entrapment than those generated from conventional feed-line and thin-film methods [31]. Different carbohydrate carriers were studied and the carrier that produces free-flowing granules (PLs) was selected. Mannitol-based PLs were spherical and smooth compared to sorbitol or microcrystalline cellulose-based ones [28]. Consequently, mannitol was adopted as a carrier to support the lipid phase in this study.

### 3.2. Analysis of Factorial Design

**Figure 1:**
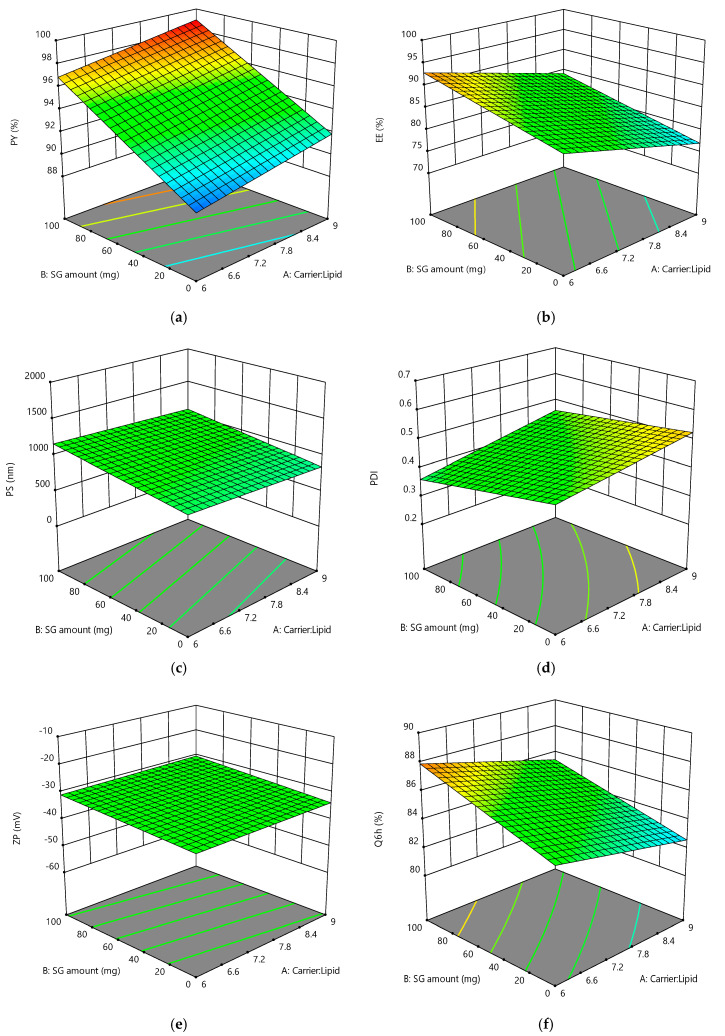
Three-dimensional response surface plots presenting the influence of the independent factors on (**a**) PY%, (**b**) EE%, (**c**) PS, (**d**) PDI, (**e**) ZP, and (**f**) Q6h.

Full factorial design is suitable to study the impact of all the factors simultaneously on the measured responses, as one can quantify the impact of the factors and their interactions on the measured responses [41]. The results of the measured responses are represented in Table 2. The selected model for each measured response was the one suggested by the design software. The significant and non-significant terms of the tested responses were differentiated, as mentioned in Table 3. The *p*-values less than 0.05 were recognized as significant, while other *p*-values higher than 0.05 were considered non-significant terms [42]. Figure 1 presents the 3-D response surface plots displaying the influence of the studied factors on the measured responses.

The R^2^ values were found to be high and close to 1 (Table 3). These high values indicate a good correlation between both actual and predicted values of the measured responses (Figure S1 in Supplementary Materials). It was observed that the predicted R^2^ values were in reasonable agreement with the adjusted R^2^ values since the difference between them was less than 0.2, as suggested by the design software, except in Y_4_ and Y_6_ responses for PDI and Q6h, respectively, (Table 3). The negative predicted R^2^ values of PDI and Q6h implied that the overall mean may be a better predictor of these responses than the current model. This might be due to the fact that PDI and Q6h of the prepared PLs were not affected by studied factors. The adequate precision measures the signal to noise ratio, it was observed that it was greater than 4 in all responses and is desirable [43] (Table 3). Thus, the selected models could be accurately used for the prediction of the measured responses and navigation of the design space [44].

#### 3.2.1. The Effect of Independent Variables on Product Yield (PY%; Y_1_)

**Figure 2:**
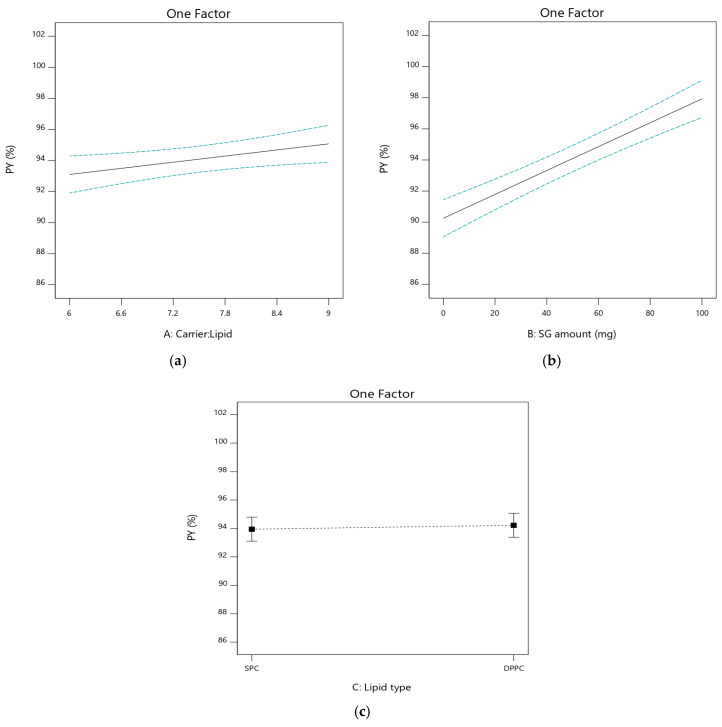
Influence of the independent factors on Y_1_ response (PY%). (**a**) Effect of A at medium level of B and C. (**b**) Effect of B at medium level of A and C. (**c**) Effect of C at medium level of A and B.

The PY% was determined after the complete drying of the prepared PLs. The PY% was in the range of 88.6 ± 2.19% to 98.8 ± 0.45%, as shown in Table 2. The impact of the independent factors on PY% is graphically illustrated in Figure 2. It was obvious that carrier/lipid phase ratio (A) had a significant impact on PY% (*p* < 0.05). There was a significant increase in PY% from 88.6 ± 2.19% to 92.7 ± 2.83% when the carrier/lipid phase ratio increased from 6:1 to 9:1 for PL2 and PL1, respectively. Similar results were reported by Omer et al. [28], who demonstrated that the PY% decreased when increasing the lipid content, due to the adherence of lipids to the flask wall.

The amount of SG (B) had a significant effect on PY% (*p* < 0.05). The PY% for formulations containing SG was 98.5 ± 1.74%, 97.3 ± 0.77%, 98.8 ± 0.45%, and 97.1 ± 1.15% for PL5, PL6, PL7, and PL8, respectively, compared to the corresponding SG-free formulations: PL1 (92.7 ± 2.83%), PL2 (88.6 ± 2.19%), PL3 (91.7 ± 1.93%), and PL4 (90.2 ± 2.61%), respectively. SG can enhance the interaction between phospholipids and mannitol, resulting in a more uniform coating and formation of PLs. This uniform coating reduces the adherence of lipids to the flask, minimizing the material loss.

Phospholipid type (C) had a non-significant effect on PY% (*p* > 0.05) as clear in PL1 (92.7 ± 2.83%) and its corresponding formulation PL3 (91.7 ± 1.93%); the same was seen for PL2 (88.6 ± 2.19%) and its corresponding formulation PL4 (90.2 ± 2.61%).

#### 3.2.2. The Effect of Independent Variables on Entrapment Efficiency (EE%; Y_2_)

**Figure 3:**
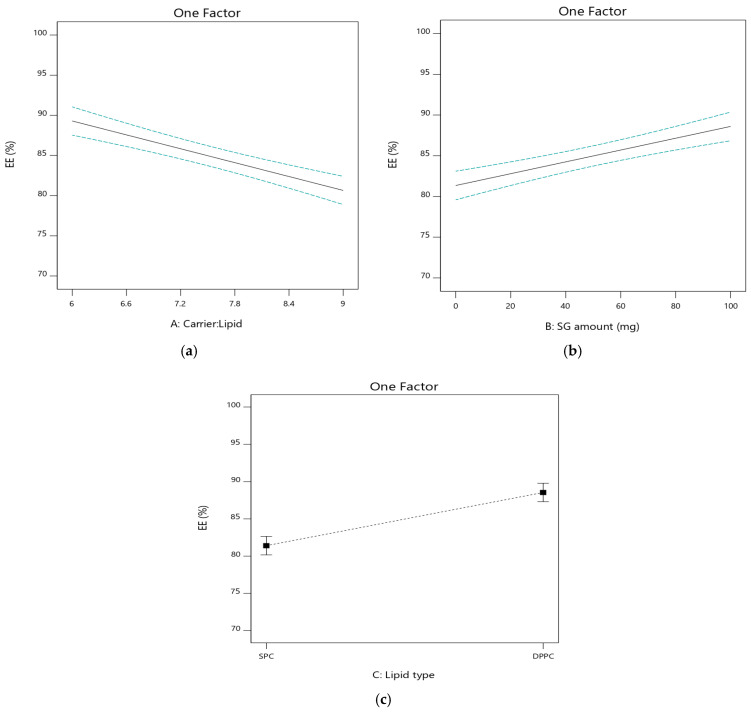
Influence of the independent factors on Y_2_ response (EE%). (**a**) Effect of A at medium level of B and C. (**b**) Effect of B at medium level of A and C. (**c**) Effect of C at medium level of A and B.

The drug was encapsulated in liposomal vesicles after aqueous hydration of the prepared PLs. The EE% of the prepared formulations ranged from 71.8 ± 2.01% to 96.1 ± 0.51%, as depicted in Table 2. The impact of the independent factors on EE% is graphically illustrated in Figure 3. It was clear that carrier/lipid phase ratio (A) had a significant influence on EE% (*p* < 0.05). When this ratio increased from 6:1 [PL2 (83.4 ± 0.81%), PL4 (88.9 ± 0.94%), PL6 (88.7 ± 1.40%), and PL8 (96.1 ± 0.51%)] to 9:1 [PL1 (71.8 ± 2.01%), PL3 (81.2 ± 2.17%), PL5 (81.7 ± 1.32%), and PL7 (87.9 ± 0.75%)] there was a significant reduction in the EE% of the corresponding formulations, respectively. The lipophilic characteristics of the drug might explain the increased EE% upon the increasing concentration of lipids. These results are consistent with previous reports revealing that increasing the total lipid concentration favors the formation of multilamellar vesicles (MLV), and thereby increases the trapped volume for the encapsulated drug within liposomes [45]. Similar results were reported by Omer et al. [28], who demonstrated that the EE% of salbutamol sulfate increased when increasing the lipid proportion in the formulation.

The percentage of drug encapsulated in liposomal vesicles may depend on many factors including liposomal size, liposome bilayers, and the effect of carbohydrate carrier on the packing pattern of liposome bilayers. There is a direct relationship between the drug leakage from the vesicles and the carrier concentration. The increase in carrier concentration resulted in a consequent decrease in the liposomal size; a small space within the liposome is available for drug entrapment that could explain the reduction in entrapment efficiency [46].

The amount of SG (B) had a significant increasing effect on EE% (*p* < 0.05). The formulations containing SG [PL5 (81.7 ± 1.32%), PL6 (88.7 ± 1.40%), PL7 (87.9 ± 0.75%), and PL8 (96.1 ± 0.51%)] had higher EE% than the corresponding SG-free formulations [PL1 (71.8 ± 2.01%), PL2 (83.4 ± 0.81%), PL3 (81.2 ± 2.17%), and PL4 (88.9 ± 0.94%)], respectively. SG creates a hydrophobic environment inside the bilayer vesicles, helping in the encapsulation and retention of hydrophobic drugs. It can also form an intermolecular hydrogen bond with IVM, helping to keep it within the liposomal vesicles and reduce its leakage.

The EE% values are also altered by varying lipid composition [47]. This variation may be a result of interaction between IVM and the head group of phospholipids with different lengths of carbon chain. The formulations based on DPPC [PL3 (81.2 ± 2.17%), PL4 (88.9 ± 0.94%), PL7 (87.9 ± 0.75%), and PL8 (96.1 ± 0.51%)] showed higher entrapment than the corresponding SPC based ones [PL1 (71.8 ± 2.01%), PL2 (83.4 ± 0.81%), PL5 (81.7 ± 1.32%), and PL6 (88.7 ± 1.40%)], respectively. It might be due to the well stabilization of liposomal membrane created by increasing the fatty acid chain length as in DPPC so that drug leakage from vesicles is reduced, leading to high entrapment values [48].

#### 3.2.3. The Effect of Independent Variables on Particle Size (PS; Y_3_)

**Figure 4:**
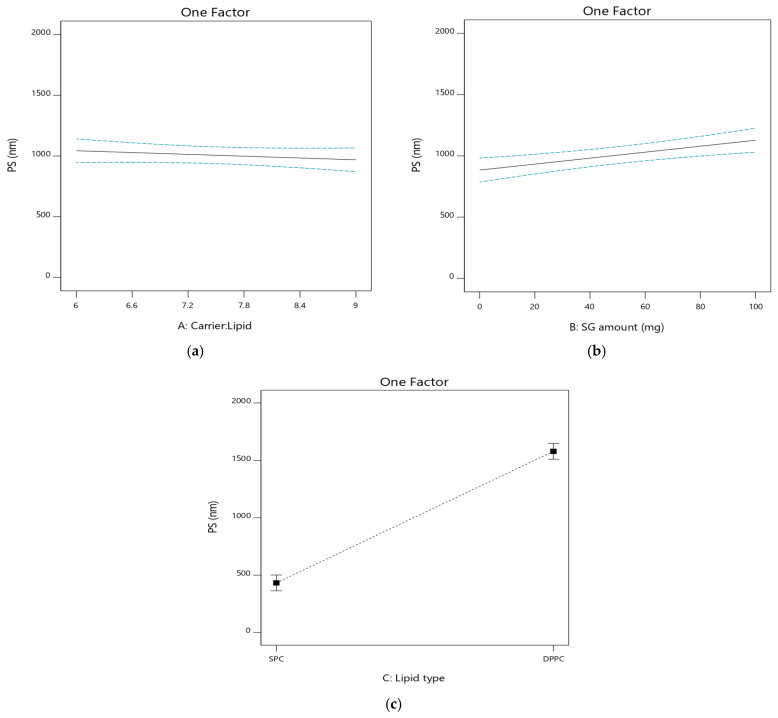
Influence of the independent factors on Y_3_ response (PS). (**a**) Effect of A at medium level of B and C. (**b**) Effect of B at medium level of A and C. (**c**) Effect of C at medium level of A and B.

Analysis of the size of liposomes was carried out upon hydration of the prepared PLs. The mean PS of the liposomal vesicles was in the range of 330.1 ± 55.65 nm to 1801.6 ± 45.61 nm, as clear in Table 2. The impact of independent factors on PS is graphically illustrated in Figure 4.

Carrier/lipid phase ratio (A) had a non-significant effect on the PS of liposomal vesicles (*p* > 0.05), as clear in PL3 (1358.3 ± 95.84 nm), and its corresponding formulation PL4 (1462.3 ± 28.86 nm); the same was true for PL5 (490.3 ± 21.75 nm) and its corresponding formulation PL6 (523.7 ± 25.86 nm).

The amount of SG (B) had a significant effect on PS (*p* < 0.05). The PS of SG-based formulations were [PL5 (490.3 ± 21.75), PL6 (523.7 ± 25.86), PL7 (1695.3 ± 46.76), and PL8 (1801.6 ± 45.61 nm)] which was higher than the corresponding SG-free formulations [PL1 (330.1 ± 55.65), PL2 (385.7 ± 49.18), PL3 (1358.3 ± 95.84), and PL4 (1462.3 ± 28.86 nm)], respectively. The increase in the size of vesicles with the addition of SG could be related to its possible placement within liposomal vesicles near the head groups of phosphatidyl choline (PC) at the interface between the glycerol groups and the nonpolar lipid chains [49]. This position resulted in an increase in the average distance among the PC molecules that constituted the bilayer of the vesicles.

Phospholipid type (C) also had a significant effect on PS (*p* < 0.05). The PS of DPPC based formulations [PL3 (1358.3 ± 95.84), PL4 (1462.3 ± 28.86), PL7 (1695.3 ± 46.76), and PL8 (1801.6 ± 45.61 nm)] were higher than the corresponding SPC based ones [PL1 (330.1 ± 55.65), PL2 (385.7 ± 49.18), PL5 (490.3 ± 21.75), and PL6 (523.7 ± 25.86 nm)], respectively (Figure S2a,b in Supplementary Materials). This may be attributed to the higher hydrophobicity of longer acyl chains in DPPC and repulsive interactions between water molecules at the interface, resulting in aggregation of vesicles [47].

#### 3.2.4. The Effect of Independent Variables on Polydispersity Index (PDI; Y_4_)

The PDI is a measure of the width of unimodal size distributions. According to the FDA, PDI is an essential parameter that should usually be considered when evaluating the liposomal preparations [27]. A value of 0 indicates homogenous dispersion, while a value of 1 indicates an entirely heterogeneous polydisperse population [43]. The mean PDI of the liposomal vesicles was in the range of 0.205 ± 0.06 to 0.603 ± 0.03 for all formulations, without extreme readings, as shown in Table 2. Factorial analysis showed that the independent variables—carrier/lipid phase ratio (A), SG amount (B), phospholipid type (C)—and their interactions showed non-significant effects on PDI with *p*-values > 0.05, as represented in Table 3.

#### 3.2.5. The Effect of Independent Variables on Zeta Potential (ZP; Y_5_)

**Figure 5:**
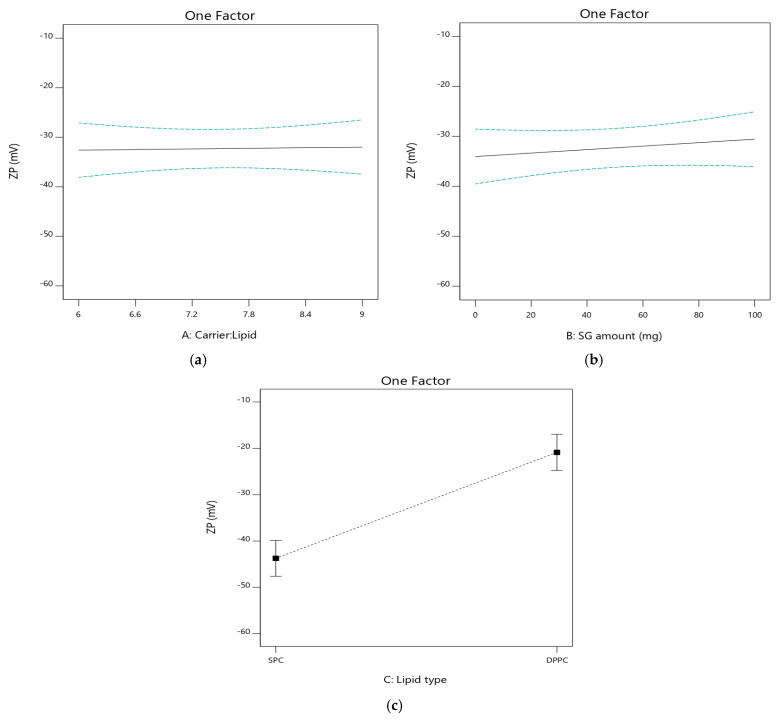
Influence of the independent factors on Y_5_ response (ZP). (**a**) Effect of A at medium level of B and C. (**b**) Effect of B at medium level of A and C. (**c**) Effect of C at medium level of A and B.

The ZP is the electrostatic charge of the particle surface; it has a major role in determining the colloidal stability by a repulsive energy barrier which inhibits particles aggregation. The ZP of all formulations is determined after hydration and the results are summarized in Table 2. The average of ZP was found to be in the range of −18.2 ± 0.60 mV to −50.1 ± 1.80 mV. Liposomal vesicles exhibit negative potential irrespective of formulation composition due to the negative charge of the polar head group of the phospholipids. A high negative net charge prevents vesicles aggregation, achieving homogenous suspension [37]. The impact of the independent factors on ZP is graphically illustrated in Figure 5.

It is clear from the results that both carrier/lipid phase ratio (A) and SG amount (B) had a non-significant effect on ZP of liposomal vesicles (*p* > 0.05). On the other hand, phospholipid type (C) influenced ZP significantly (*p* < 0.05). The ZP (as an absolute value) of DPPC based formulations [PL3 (−25.4 ± 1.35), PL4 (−18.2 ± 0.60), PL7 (−20.1 ± 1.40), and PL8 (−19.7 ± 0.73 mV)] were lower than those of the corresponding formulations with SPC [PL1 (−42.4 ± 4.80), PL2 (−50.1 ± 1.80), PL5 (−40.1 ± 1.04) and PL6 (−42.4 ± 1.10 mV)], respectively (Figure S2c,d in Supplementary Materials). The negative charge was greater in case of SPC-based formulations because SPC contains unsaturated fatty acids, the carboxylic acid groups of these unsaturated fatty acids potentially increasing the negative charge.

#### 3.2.6. The Effect of Independent Variables on Drug Released After 6 h (Q6h; Y_6_)

Reconstituted liposomes were subjected to an in vitro release investigation across a dialysis membrane in order to determine the impact of the investigated independent factors on the drug release. The pH of the dissolution medium was selected to simulate the pH of alveoli; the conducting airways are lined with a mucus gel-aqueous sol complex of up to 100 µm in depth called air surface liquid (ASL), the pH of this mucosal layer is acidic compared to blood pH [50]. Sodium lauryl sulfate was added to enhance the release of IVM, considering the ability of surfactants to accelerate this process due to the reduction in the interfacial tension and micellar solubilization properties [10]. Drug release after 6 h (Q6h%) of the prepared formulations ranged from 80.95 ± 1.36% to 88.79 ± 2.03%, as depicted in Table 2. As clear from the results, the studied factors and their interactions had non-significant effects on Q6h, with *p*-values > 0.05, as represented in Table 3.

The release profiles for all the developed PLs exhibited relatively similar drug release characteristics at the end of 6 h (Figure S3 in Supplementary Materials), a typical biphasic pattern was observed with an initial rapid phase followed by a slow sustained phase. The burst release of the drug due to the presence of unentrapped drug in the outer layers of liposomes is expected to be responsible for the initial rapid phase [51]. The PLs have the ability to convert the physical nature of the loaded lipophilic drugs from the crystalline state into an amorphous state, resulting in an increased solubility of the entrapped lipophilic drugs inside PLs. In addition, the hydrophilic nature of the mannitol which facilitates the quick hydration of PLs to transform into liposomes might be one of the causes for enhancing the drug release [25].

#### 3.2.7. Kinetic Study of Drug Release

IVM release mechanism from PLs formulations was studied using in vitro release data from various mathematical models, as shown in Table 4. The model that had the highest R^2^ value was Korsmeyer–Peppas model, making it the most suitable option for describing the process of IVM release. The Korsmeyer–Peppas model n value was less than 0.5 for all formulations. This could indicate the fitness of drug release to quasi-Fickian diffusion mechanism (case I transport) [52].

### 3.3. Optimization Technique

The aim of the optimization step was to achieve the maximum PY%, EE%, ZP (as absolute value), and Q6h and the minimum PS and PDI, as previously prescribed in Table 1. The optimized formulation suggested by the design software was PL6, which met these criteria with a high desirability value (0.830). The predicted and observed responses of PL6 were compared as shown in Table 5 to validate the experiment. The models’ validity and the design’s good predictability were confirmed by the high correlation between the observed and predicted values [36]. The aim of this study is to investigate the activity of IVM-loaded PLs and SG modified IVM-loaded PLs in IBV-infected ECEs. Subsequently, PL6 and its corresponding formulation (PL2) were selected as the optimized formulations for further investigations.

### 3.4. Characterization of the Selected IVM-Loaded PLs Formulations

#### 3.4.1. Scanning Electron Microscopy (SEM)

**Figure 6:**
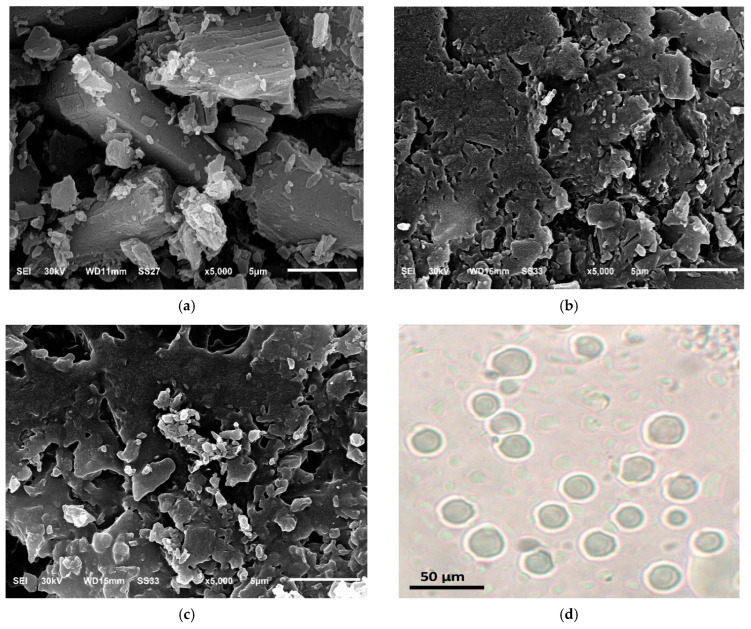
Scanning electron micrographs (**a**) mannitol, (**b**) PL2, (**c**) PL6 at 5000×, and (**d**) photomicrograph of liposomal dispersion which formed upon hydration of DPPC based PLs.

The SEM photographs revealed that the surface morphology of mannitol in the selected formulations (PL2 and PL6) was different from pure mannitol. The crystalline form of mannitol which presented in its pure sample was not clear in the selected formulations due to deposition of phospholipid on its surface [33]. Observation under digital microscope revealed that the proliposomal particles were rapidly converted to liposomes within a few minutes following contact with water, as presented in Figure 6c.

#### 3.4.2. DSC Analysis

**Figure 7:**
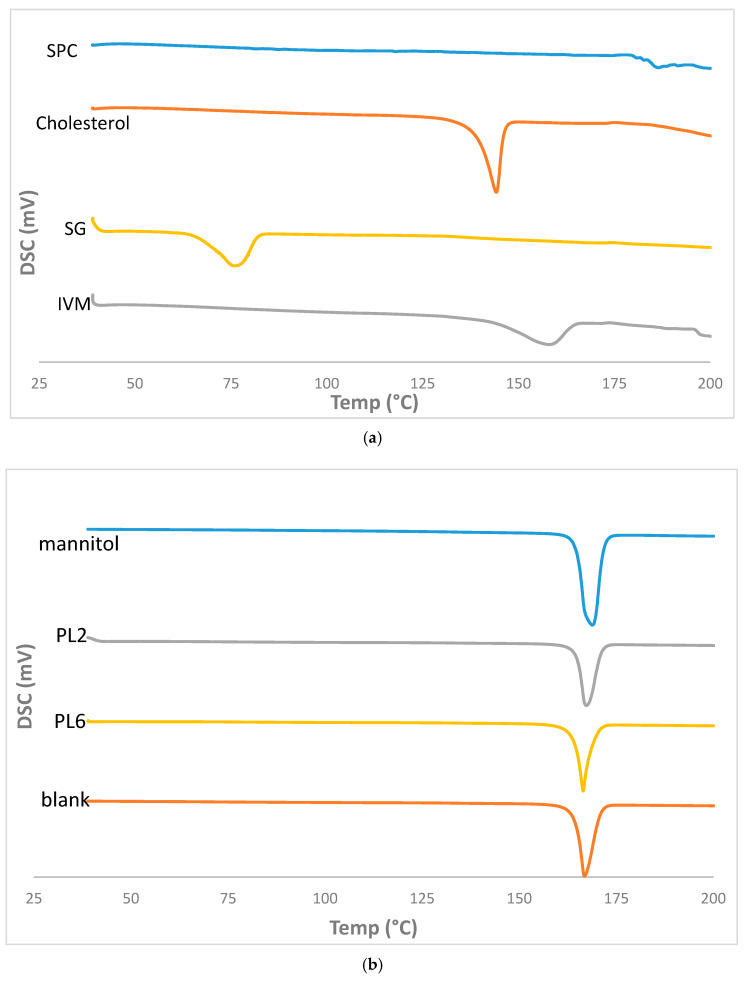
(**a**,**b**) DSC thermograms of pure IVM, SG, cholesterol, SPC, mannitol, selected formulations (PL2 and PL6), and the blank formulation.

The physical state of the drug in the proliposomal formulation was studied using DSC, where any shift or disappearance of peaks could indicate the alteration in the crystallinity of the drug [34,53]. The DSC thermograms of pure IVM, SG, SPC, cholesterol, mannitol, selected formulations (PL2 and PL6), and the blank formulation are illustrated in Figure 7a,b. Pure IVM and SG showed endothermic peaks at 158.69 °C and 76.69 °C, respectively, corresponding to their crystalline state at their melting point temperature [54,55]. The characteristic peak of SPC was at 187.42 °C, corresponding to its melting temperature (transition from gel state to liquid crystal state) [42]. The characteristic peak of cholesterol was at 144.47 °C. The sharp characteristic peak of mannitol was at 167.66 °C, corresponding to its crystalline state at this melting point [25]; this peak was preserved in the thermograms of the blank formulation and selected formulations (PL2 and PL6), signifying the maintenance of the crystalline state of mannitol in PLs formulations [25]. The DSC thermograms of the selected formulations showed no representative peak of IVM, suggesting its transformation from crystalline state to amorphous form or the dissolution of the drug in the molten mass [25].

#### 3.4.3. FTIR

**Figure 8:**
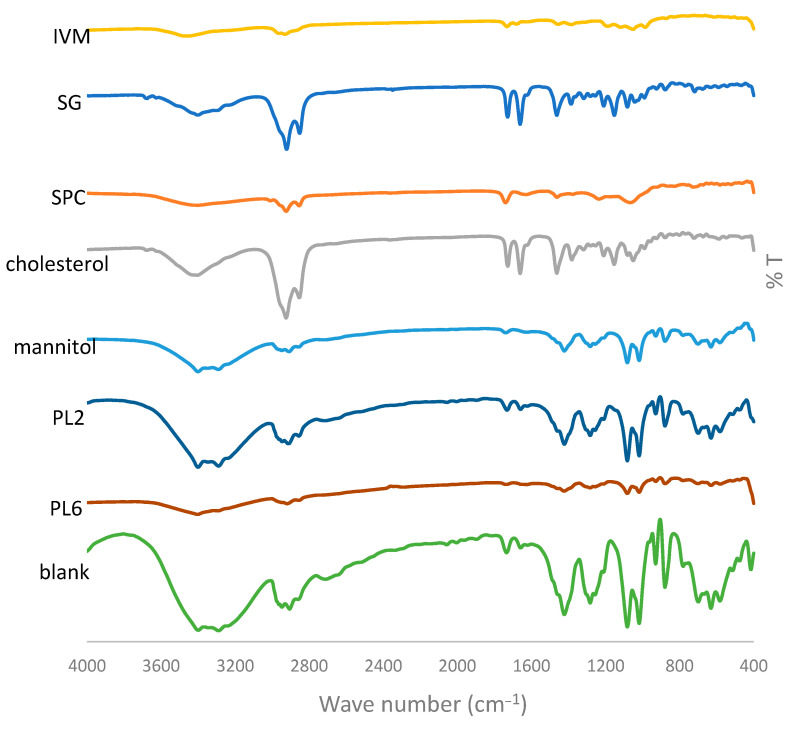
FTIR spectra of pure IVM, SG, SPC, cholesterol, mannitol, the selected formulations (PL2 and PL6), and the blank formulation.

FTIR analysis was conducted to investigate the possible interactions between IVM and excipients based on characteristic drug peaks’ absence or shifts [25,33]. The FTIR spectra of pure IVM, SG, SPC, cholesterol, mannitol, PL2, PL6, and the blank formulation are presented in Figure 8. FTIR spectrum of pure IVM displayed characteristic bands at 3450.99 cm^−1^ (O-H stretching), 2931.27 cm^−1^ (C-H stretching), 1732.73 cm^−1^ and 1690.66 cm^−1^ (C=O stretching), 1458.96 cm^−1^ (O-H bending), and 1394.64 cm^−1^ (C-O stretching). The FTIR spectrum of mannitol showed characteristic peaks at 3290.83 cm^−1^ (O-H stretching of alcohol), 1082.83 and 1019.23 cm^−1^ (C-O stretching of alcohol), 929.521 cm^−1^ and 879.381 cm^−1^ (C-H out of plane bending). The FTIR spectrum of SPC showed characteristic peaks at 2924.52 cm^−1^ and 2855.1 cm^−1^ (stretching vibration absorption of CH2), 1740.44 cm^−1^ (C=O stretching vibration of carboxylic acid). Cholesterol showed principal absorption peaks at 2925.48 cm^−1^, corresponding to the C-H aliphatic stretching and 1402.74 cm^−1^, corresponding to the plane bending for CH_3_, OH. On comparing the FTIR spectrum of blank formulation and these of the selected formulations, the results confirmed the absence of additional peaks and no chemical interaction between IVM and other components of PLs [25,56]. This was in great alignment with what was found by Janga et al. [51].

#### 3.4.4. Stability Studies

**Figure 9:**
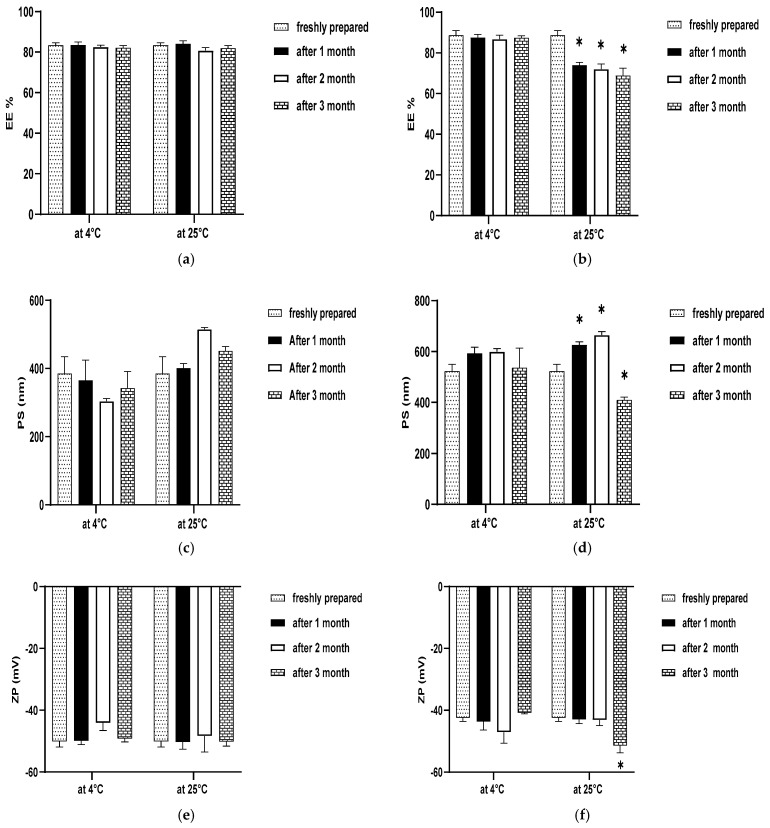
Outline of stability studies for selected formulations for 1, 2, and 3 months at 4 ± 1 °C and 25 ± 1 °C in terms of (**a**) EE% of PL2, (**b**) EE% of PL6, (**c**) PS of PL2, (**d**) PS of PL6, (**e**) ZP of PL2, and (**f**) ZP of PL6. * Significant (*p* < 0.05).

The results of the stability studies for the selected formulations (PL2 and PL6) are presented in Figure 9. No signs of crystallization were observed under an optical microscope, the PLs were free flowing without any agglomeration when detected visually and the formation of the liposomal vesicles was rapid upon hydration. For PL2 there was a non-significant difference (*p* > 0.05) in the EE%, PS, and ZP for samples stored at 4 ± 1 °C and 25 ± 1 °C compared to the freshly prepared one at all time intervals (1, 2 and 3 months), which indicates its stability in storage at room temperature and refrigerated conditions.

There was a non-significant difference (*p* > 0.05) in the EE%, PS, and ZP for PL6 stored at 4 ± 1 °C compared to the freshly prepared one at all time intervals (1, 2 and 3 months). On the other hand, the differences in PS and EE% were significant at each time interval, and in ZP at 3 months only for samples stored at 25 ± 1 °C; this significant difference may be related to SG as it requires lower temperatures to be stable. So, we recommend storing the prepared formulations in refrigerated conditions.

### 3.5. Investigation of the Anti-Viral Activity Against IBV

#### 3.5.1. Toxicity Study

**Figure 10:**
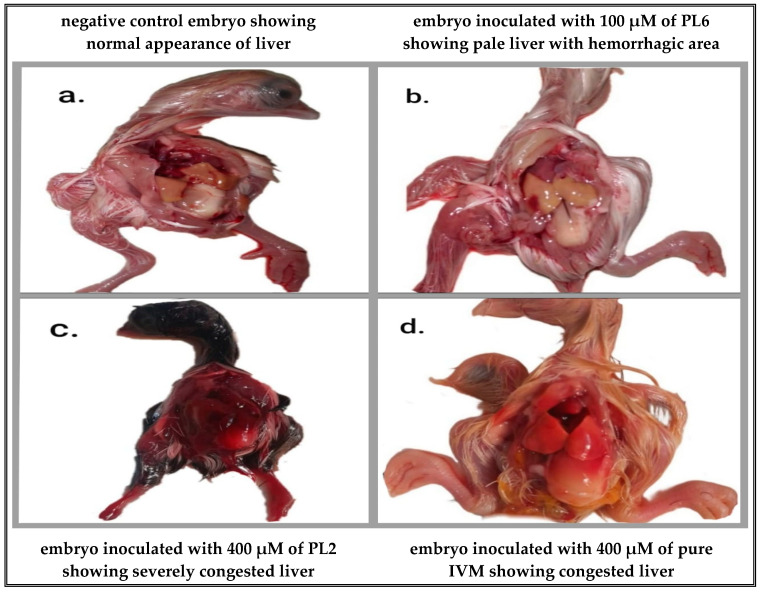
Macroscopic lesions of embryos in negative control and drug-inoculated groups. (**a**) Negative control embryo showing normal appearance of liver. (**b**) Embryo inoculated with 100 µM of PL6 showing pale liver with hemorrhagic area. (**c**) Embryo inoculated with 400 µM of PL2 showing severely congested liver. (**d**) Embryo inoculated with 400 µM of pure IVM showing congested liver.

The MNTC was calculated for pure IVM, PL2, and PL6 in ECEs. It was identified as the concentration that yielded more than 50% survival or that exhibited signs of lesions not exceeding 50% of inoculated embryos. Macroscopic lesions of embryos are shown in Figure 10. For pure IVM, ECEs inoculated with 400 µM and 200 µM showed 66.7% and 33.3% survival, with 100% lesions in both concentrations. Meanwhile, ECEs inoculated with 100 µM showed 33.3% lesions and 100% survival. Absence of death and lesions were observed in ECEs inoculated with lower concentrations. ECEs inoculated with PL2 exhibited 100% and 66.7% survival for those that received 400 µM and 200 µM, respectively, with 100% and 33.3% lesions, respectively, and the absence of death and lesions in ECEs inoculated with lower concentrations. It was observed that the liposomal vesicles of IVM has lower toxicity in ECEs than pure IVM, and this result was in accordance with Croci et al. [6]. Drugs encapsulated in liposomes are released gradually and in a controlled manner. This avoids the sudden increase in drug concentration in the bloodstream that often occurs with free drugs, thereby reducing the risk of toxicity. ECEs inoculated with 400 µM and 200 µM of PL6 exhibited 66.7% for both survival and lesions. Meanwhile, ECEs inoculated with 100 µM showed 33.3% lesions and 100% survival and there was no death and lesions in ECEs inoculated with further lower concentrations.

#### 3.5.2. Assay of Anti-Viral Activity

**Figure 11:**
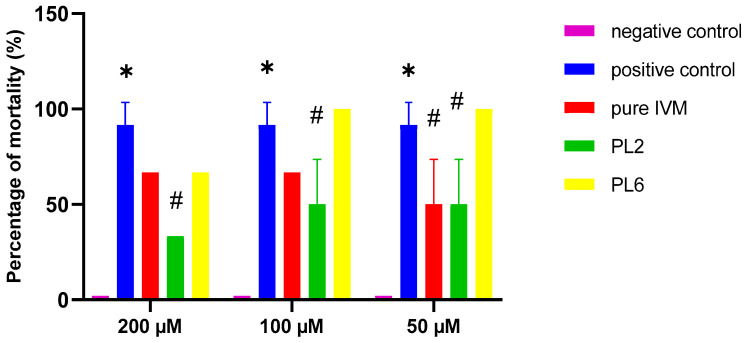
Influence of pure IVM, PL2, and PL6 in the percentage of mortality of IBV-infected ECEs. The positive control group included 10^3^ EID50 per 100 µL of IB MA5 virus. Data are the result of two independent experiments performed in duplicate. Statistical significance was assessed by two-way analysis of variance (ANOVA) with Tukey post hoc test. Values are expressed as mean ± standard error of mean. * *p* < 0.05 in comparison to negative control group; # *p* < 0.05 in comparison to positive control group.

The mortality percentage of ECEs was calculated five days post-inoculation and the results were analyzed using GraphPad Prism version 8. As presented in Figure 11, the positive control group showed a significantly high percentage of mortality in comparison with the negative control group after five days of inoculation with IBV. There is a reduction in the percentage of mortality of IBV-infected ECEs inoculated with pure IVM in comparison with positive control group and this reduction was significant at 50 µM (*p* < 0.05).

Inoculation with PL2 resulted in a greater reduction in the percentage of mortality of IBV-infected ECEs compared with those inoculated with pure IVM at all studied doses, this may be due to high cellular uptake of liposomal vesicles of IVM in comparison with free IVM. It was also observed that there was a significant reduction in the percentage of mortality of IBV-infected ECEs at all studied doses of PL2 compared with the positive control group (*p* < 0.05).

On the other hand, there was a non-significant difference in the percentage of mortality of PL6-inoculated IBV-infected ECEs compared with the positive control group (*p* > 0.05). Impaired fitting on the receptor due to masking the functional group of IVM by the steric hindrance of SG may be the reason for the higher mortality percentage in PL6 receiving, IBV-infected ECEs compared with that in PL2-receiving, IBV-infected ECEs.

**Figure 12:**
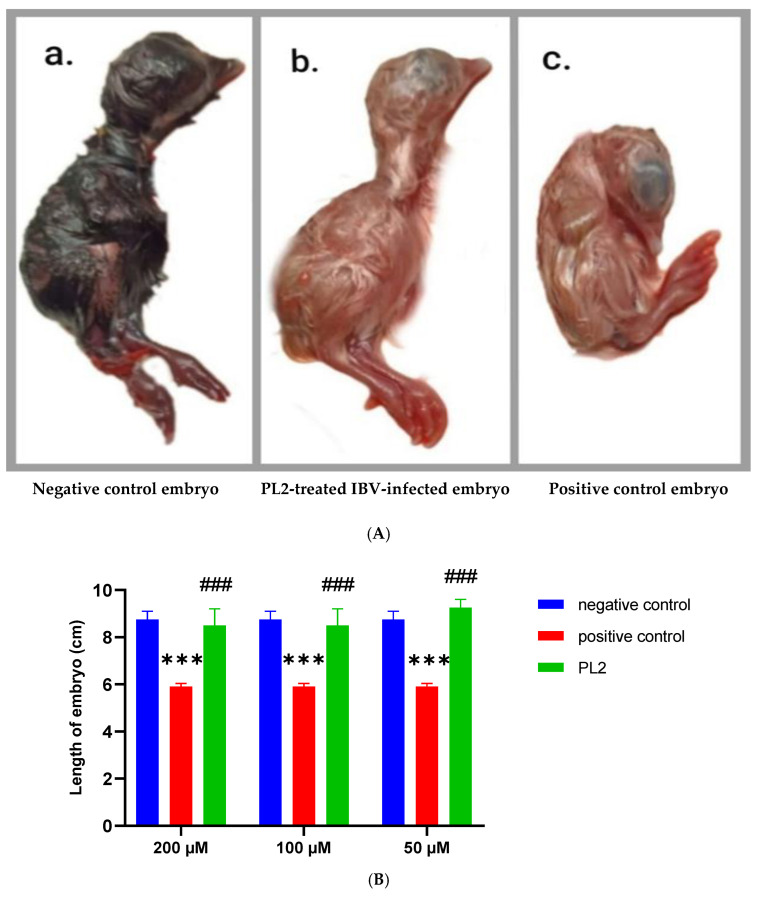
(**A**) Macroscopic visualization of embryos in negative control, IBV-infected and virus-treated inoculated ECEs. [(**a**) Uninoculated control embryo. (**b**) IBV-infected embryo treated with 200 µM of PL2. (**c**) Curling and dwarfing in embryo inoculated with IBV only]. (**B**) The lengths of embryos in negative control, IBV-infected and virus-treated inoculated ECEs. The positive control group included 10^3^ EID50 per 100 µL of IB MA5 virus. Data are the result of two independent experiments performed in duplicate. Statistical significance was assessed by two-way analysis of variance (ANOVA) with Tukey post hoc test. Values are expressed as mean ± standard error of mean. *** *p* < 0.001 in comparison to negative control group and ### *p* < 0.001 in comparison to positive control group.

Macroscopic visualization of the embryos showed an absence of curling and dwarfing in IBV-infected ECEs treated with PL2, as presented in Figure 12A; there was a statistical significant difference (*p* < 0.001) in the lengths of ECEs in the positive control group compared to those of the negative control group. On the other hand, it was observed that the lengths of IBV-infected ECEs treated with PL2 at all studied doses were not significantly differed from those of the negative group, but there was a statistically significant difference (*p* < 0.001) in comparison with those of the positive control group: Figure 12B.

**Figure 13:**
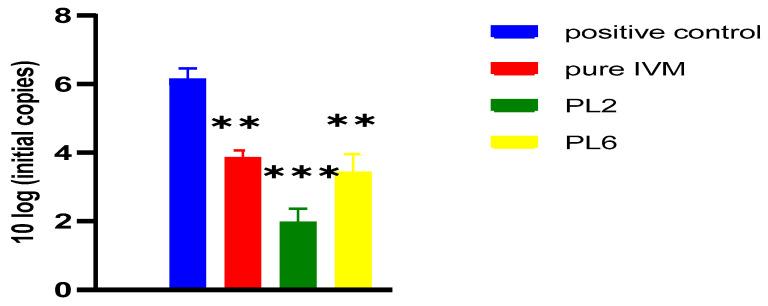
Influence of 200 µM of pure IVM, PL2, and PL6 in the viral load of IBV-infected ECEs 72 h post inoculation. The positive control group included 10^3^ EID50 per 100 µL of IB MA5 virus. Data are the result of two independent measurements. Statistical significance was assessed by one-way analysis of variance (ANOVA) with Tukey post hoc test. Values are expressed as mean ± standard error of mean. *** *p* < 0.001; ** *p* < 0.01 in comparison to positive control group.

In addition, the viral load was quantified (Figure 13); there is a significant reduction in the copies of IBV 72 h post-inoculation in the pure IVM-treated group in comparison with the positive control group (*p* < 0.01). The group treated with PL2 exhibited further reduction in IBV’s copies (*p* < 0.05) in comparison with IVM-treated group and this reduction was more significant when compared to positive control group (*p* < 0.001). On the other hand, there is a non-significant difference in copies of IBV in the PL6-treated group in comparison with the pure IVM-treated group, but this difference was significant when compared to the positive control group (*p* < 0.01).

## 4. Conclusions

In this study, IVM-loaded PLs were fabricated, characterized, and optimized using a 2^3^ full factorial design. Furthermore, the selected formulations were investigated for their efficacy against IBV. All of the prepared formulations exhibited high EE%, ranging from 71.8 ± 2.01% to 96.1 ± 0.51%, adequate repulsive force to improve physical liposomal stability, and cumulative release values ranging from 80.95 ± 1.36% to 88.79 ± 2.03%. For three months, the chosen formulations demonstrated good stability. Additionally, research into the anti-viral action against IBV-infected ECEs revealed a much lower mortality rate for ECEs with PL2 inoculations compared to IBV-infected ECEs that did not receive any treatment (positive control). These findings demonstrated that loading IVM into PLs increased IVM’s anti-viral action against IBV.

## Figures and Tables

**Table 1:** Selected independent factors, dependent responses, and the criteria set (goals) for selecting the optimized formulation.

Independent Factors	Factors Symbol	Unit	Factors Type	Factors Levels	
				Low (−1)	High (+1)
Carrier/lipid phase (*w*/*w*)	A	---	Numeric	6:1	9:1
Amount of SG	B	mg	Numeric	Zero	100
phospholipid type	C	---	Categoric	SPC	DPPC
**Dependent Responses**	**Response Symbol**	**Unit**	**(Goals)**		
Product yield	Y_1_	%	Maximize		
Entrapment efficiency	Y_2_	%	Maximize		
Particle size	Y_3_	nm	Minimize		
Polydispersity index	Y_4_	---	Minimize		
Zeta potential	Y_5_	mV	Maximize (as absolute value)		
Cumulative percentage of drug release after 6 h	Y_6_	%	Maximize		

Abbreviations: SG—stearyl glycyrrhetinate; SPC—soybean phosphatidylcholine; DPPC—dipalmitoyl phosphatidylcholine.

**Table 2:** Eight experimental runs, as suggested by 2^3^ factorial design, and observed values of the measured responses.

Formulation Code	A	B	C	PY% (Y_1_)	EE% (Y_2_)	PS (nm) (Y_3_)	PDI (Y_4_)	ZP (mV) (Y_5_)	Q6h (Y_6_)
PL1	9:1	Zero	SPC	92.7 ± 2.83	71.8 ± 2.01	330.1 ± 55.65	0.603 ± 0.03	−42.4 ± 4.80	80.95 ± 1.36
PL2	6:1	Zero	SPC	88.6 ± 2.19	83.4 ± 0.81	385.7 ± 49.18	0.585 ± 0.04	−50.1 ± 1.80	86.59 ± 3.01
PL3	9:1	Zero	DPPC	91.7 ± 1.93	81.2 ± 2.17	1358.3 ± 95.84	0.447 ± 0.02	−25.4 ± 1.35	84.23 ± 4.56
PL4	6:1	Zero	DPPC	90.2 ± 2.61	88.9 ± 0.94	1462.3 ± 28.86	0.327 ± 0.01	−18.2 ± 0.60	82.49 ± 1.69
PL5	9:1	100	SPC	98.5 ± 1.74	81.7 ± 1.32	490.3 ± 21.75	0.396 ± 0.06	−40.1 ± 1.04	84.59 ± 4.19
PL6	6:1	100	SPC	97.3 ± 0.77	88.7 ± 1.40	523.7 ± 25.86	0.205 ± 0.06	−42.4 ± 1.10	88.79 ± 2.03
PL7	9:1	100	DPPC	98.8 ± 0.45	87.9 ± 0.75	1695.3 ± 46.76	0.528 ± 0.08	−20.1 ± 1.40	86.31 ± 1.45
PL8	6:1	100	DPPC	97.1± 1.15	96.1 ± 0.51	1801.6 ± 45.61	0.517 ± 0.05	−19.7 ± 0.73	87.02 ± 1.88

Abbreviations: A—carrier to lipid phase ratio (*w*/*w*); B—SG amount (mg); C—phospholipid type. Data represented as mean ± SD (n = 3). PY%—product yield percentage; EE%—entrapment efficiency percentage; PS—particle size; PDI—polydispersity index; ZP—zeta potential; Q6h—cumulative percentage of drug released after 6 h.

**Table 3:** ANOVA statistical analysis of responses (Y_1_–Y_6_).

Suggested Model	Y_1_ (%)	Y_2_ (%)	Y_3_ (nm)	Y_4_	Y_5_ (mV)	Y_6_ (%)
	Linear	Linear	Linear	2-FI	Linear	2-FI
	*p*-Value	*p*-Value	*p*-Value	*p*-Value	*p*-Value	*p*-Value
Model	0.0011 *	0.0006 *	<0.0001 *	0.4922	0.0056 *	0.3698
A	0.0216 *	0.0007 *	0.2073	0.4408	0.8404	0.2647
B	0.0003 *	0.0013 *	0.0081 *	0.4638	0.2842	0.1928
C	0.7780	0.0014 *	<0.0001 *	0.9325	0.0012 *	0.8599
AB	---	---	---	0.8579	---	0.8383
AC	---	---	---	0.8282	---	0.2188
BC	---	---	---	0.2022	---	0.8756
Significant factors	A, B	A, B, C	B, C	----	C	----
**R^2^ Analysis**						
R^2^	0.9762	0.9821	0.9929	0.9237	0.9451	0.9589
Adequate precision	16.2385	25.5932	29.4301	4.2675	9.6516	6.0941
Adjusted R^2^	0.9584	0.9688	0.9875	0.4659	0.9039	0.7124
Predicted R^2^	0.9049	0.9286	0.9715	−3.8833	0.7803	−1.6297

Abbreviations: Y_1_—product yield (PY); Y_2_—entrapment efficiency (EE); Y_3_—particle size (PS); Y_4_—polydispersity index (PDI); Y_5_—zeta potential (ZP); Y_6_—cumulative percentage of drug released after 6 h (Q6h); A—carrier/lipid ratio (*w*/*w*); B—SG amount (mg); C—phospholipid type. * Significant (*p* < 0.05).

**Table 4:** In vitro release kinetics data of the prepared PLs formulations.

Release Model	R^2^							
	PL1	PL2	PL3	PL4	PL5	PL6	PL7	PL8
Zero order	0.1912	0.2646	0.2565	0.1951	0.0545	0.0737	0.0860	0.0536
First order	0.8923	0.9381	0.9070	0.9100	0.9019	0.9403	0.9303	0.9257
Higuchi	0.8727	0.9009	0.9012	0.8547	0.8131	0.8174	0.8122	0.8021
Korsmeyer–Peppas	0.9612	0.9722	0.9762	0.9363	0.9387	0.9365	0.9236	0.9246
Hixson–Crowell	0.7961	0.8662	0.8153	0.8312	0.8286	0.8886	0.8784	0.8710
Best fit model	Korsmeyer–Peppas	Korsmeyer–Peppas	Korsmeyer–Peppas	Korsmeyer–Peppas	Korsmeyer–Peppas	First order	First order	First order
n-value of Korsmeyer–Peppas	0.312	0.328	0.324	0.318	0.283	0.287	0.293	0.284

**Table 5:** Predicted and observed values for the optimized formulation (PL6).

	Y_1_ (%)	Y_2_ (%)	Y_3_ (nm)	Y_4_	Y_5_ (mV)	Y_6_ (%)
Predicted values	96.775	89.35	591.675	0.240	−42.325	89.276
Observed values	97.3	88.7	523.7	0.205	−42.4	88.79

Abbreviations: Y_1_—product yield (PY); Y_2_—entrapment efficiency (EE); Y_3_—particle size (PS); Y_4_—polydispersity index (PDI); Y_5_—zeta potential (ZP); Y_6_—cumulative percentage of drug released after 6 h (Q6h).

## Data Availability

The data presented in this study are available in this article and the Supplementary Materials.

## Supplementary Materials

The following supporting information can be downloaded at: https://www.mdpi.com/article/10.3390/pharmaceutics17020165/s1, Figure S1: Linear correlation plots between actual and predicted values for (a) Y_1_ response, (b) Y_2_ response, (c) Y_3_ response, (d) Y_4_ response, (e) Y_5_ response, (f) Y_6_ response. Figure S2: PS and ZP of IVM-loaded PLs formulations. (a) PS of SPC based IVM-Loaded PLs, (b) PS of DPPC based IVM-Loaded PLs, (c) ZP of SPC based IVM-Loaded PLs, (d) ZP of DPPC based IVM-Loaded PLs. Figure S3: In vitro drug release profiles of IVM-loaded PLs formulations (n = 3 ± SD).

## References

[1] Cavanagh D. (2007). Coronavirus Avian Infectious Bronchitis Virus. Vet. Res..

[2] Ignjatovic J., Sapats S. (2000). Avian Infectious Bronchitis Virus. Rev. Sci. Tech..

[3] Bhuiyan S.A., Amin Z., Rodrigues K.F., Saallah S., Shaarani S., Sarker S., Siddiquee S. (2021). Veterinary Sciences Infectious Bronchitis Virus (Gammacoronavirus) in Poultry Farming: Vaccination, Immune Response and Measures for Mitigation. Vet. Sci..

[4] Hellwig M.D., Maia A. (2020). A COVID-19 Prophylaxis? Lower Incidence Associated with Prophylactic Administration of Ivermectin. Int. J. Antimicrob. Agents.

[5] Popp M., Reis S., Schießer S., Hausinger R.I., Stegemann M., Metzendorf M.-I., Kranke P., Meybohm P., Skoetz N., Weibel S. (2022). Ivermectin for Preventing and Treating COVID-19 (Review). Cochrane Database Syst. Rev..

[6] Croci R., Bottaro E., Chan K.W.K., Watanabe S., Pezzullo M., Mastrangelo E., Nastruzzi C. (2016). Liposomal Systems as Nanocarriers for the Antiviral Agent Ivermectin. Int. J. Biomater..

[7] Mahnashi M.H., Mahmoud A.M., Alkahtani S.A., El-wekil M.M. (2021). Ivermectin Detection Using Ag@ B, S Co-Doped Reduced Graphene Oxide Nanohybrid. J. Alloys Compd..

[8] Crump A., Ōmura S. (2011). Ivermectin, ‘Wonder Drug’ from Japan: The Human Use Perspective. Proc. Jpn. Acad..

[9] Awad H., Rawas-Qalaji M., El Hosary R., Jagal J., Ahmed I.S. (2023). Formulation and Optimization of Ivermectin Nanocrystals for Enhanced Topical Delivery. Int. J. Pharm. X.

[10] Starkloff W.J., Bucalá V., Palma S.D., Vidal N.L.G. (2017). Design and in Vitro Characterization of Ivermectin Nanocrystals Liquid Formulation Based on a Top-down Approach. Pharm. Dev. Technol..

[11] Varghese F.S., Kaukinen P., Gläsker S., Bespalov M., Hanski L., Wennerberg K., Kümmerer B.M., Ahola T. (2016). Discovery of Berberine, Abamectin and Ivermectin as Antivirals against Chikungunya and Other Alphaviruses. Antivir. Res..

[12] Lv C., Liu W., Wang B., Dang R., Qiu L., Ren J., Yan C., Yang Z., Wang X. (2018). Ivermectin Inhibits DNA Polymerase UL42 of Pseudorabies Virus Entrance into the Nucleus and Proliferation of the Virus In Vitro and Vivo. Antivir. Res..

[13] Yang S.N.Y., Atkinson S.C., Wang C., Lee A., Bogoyevitch M.A., Borg N.A., Jans D.A. (2020). The Broad Spectrum Antiviral Ivermectin Targets the Host Nuclear Transport Importin α/Β1 Heterodimer. Antivir. Res..

[14] Caly L., Druce J.D., Catton M.G., Jans D.A., Wagstaff K.M. (2020). The FDA-Approved Drug Ivermectin Inhibits the Replication of SARS-CoV-2 In Vitro. Antivir. Res..

[15] Bryant A., Lawrie T.A., Dowswell T., Fordham E.J., Mitchell S., Hill S.R., Tham T.C. (2021). Ivermectin for Prevention and Treatment of COVID-19 Infection: A Systematic Review, Meta-Analysis, and Trial Sequential Analysis to Inform Clinical Guidelines. Am. J. Ther..

[16] Formiga F.R., Leblanc R., de Souza Rebouças J., Farias L.P., de Oliveira R.N., Pena L. (2021). Ivermectin: An Award-Winning Drug with Expected Antiviral Activity Against COVID-19. J. Control. Release.

[17] de Souza Z.C., Júnior F.H.X., Pinheiro I.O., de Souza Rebouças J., de Abreu B.O., Mesquita P.R.R., de Medeiros Rodrigues F., Quadros H.C., Mendes T.M.F., Nguewa P. (2023). Ameliorating the Antiparasitic Activity of the Multifaceted Drug Ivermectin through a Polymer Nanocapsule Formulation. Int. J. Pharm..

[18] Ali M., Afzal M., Verma M., Misra-Bhattacharya S., Ahmad F.J., Dinda A.K. (2013). Improved Antifilarial Activity of Ivermectin in Chitosan—Alginate Nanoparticles against Human Lymphatic Filarial Parasite, Brugia Malayi. Parasitol. Res..

[19] Ahmadpour E., Godrati-Azar Z., Spotin A., Norouzi R., Hamishehkar H., Nami S., Heydarian P., Rajabi S., Mohammadi M., Perez-Cordon G. (2019). Nanostructured Lipid Carriers of Ivermectin as a Novel Drug Delivery System in Hydatidosis. Parasit. Vectors.

[20] Chen B.Z., Yang Y., Wang B.B., Ashfaq M., Guo X.D. (2018). Self-Implanted Tiny Needles as Alternative to Traditional Parenteral Administrations for Controlled Transdermal Drug Delivery. Int. J. Pharm..

[21] Bailly C., Vergoten G. (2020). Glycyrrhizin: An Alternative Drug for the Treatment of COVID-19 Infection and the Associated Respiratory Syndrome?. Pharmacol. Ther..

[22] Gomaa A.A., Abdel-Wadood Y.A. (2021). The Potential of Glycyrrhizin and Licorice Extract in Combating COVID-19 and Associated Conditions. Phytomed. Plus.

[23] Selyutina O.Y., Polyakov N.E. (2019). Glycyrrhizic Acid as a Multifunctional Drug Carrier—From Physicochemical Properties to Biomedical Applications: A Modern Insight on the Ancient Drug. Int. J. Pharm..

[24] Asl M.N., Hosseinzadeh H. (2008). Review of Pharmacological Effects of *Glycyrrhiza* sp. and Its Bioactive Compounds. Phyther. Res..

[25] Gangishetty H., Eedara B.B., Bandari S. (2015). Development of Ketoprofen Loaded Proliposomal Powders for Improved Gastric Absorption and Gastric Tolerance: In Vitro and In Situ Evaluation. Pharm. Dev. Technol..

[26] Vanic Z., Planinšek O., Škalko-Basnet N., Tho I. (2014). Tablets of Pre-Liposomes Govern in Situ Formation of Liposomes: Concept and Potential of the Novel Drug Delivery System. Eur. J. Pharm. Biopharm..

[27] Abed O.S.A., Mulkala S., Sharif I., Abdin A.M., Elkordy A.A. (2021). Lyophilization-Free Proliposomes for Sustained Release Oral Delivery of Hydrophobic Drug (Cinnarazine): A Comparative Study. Pharm. Technol. Hosp. Pharm..

[28] Omer H.K., Hussein N.R., Ferraz A., Najlah M., Ahmed W., Taylor K.M.G., Elhissi A.M.A. (2018). Spray-Dried Proliposome Microparticles for High-Performance Aerosol Delivery Using a Monodose Powder Inhaler. AAPS PharmSciTech.

[29] Singh N., Kushwaha P., Ahmad U., Abdullah M. (2019). Proliposomes: An Approach for the Development of Stable Liposome. Ars. Pharm..

[30] Muneer S., Masood Z., Butt S., Anjum S., Zainab H., Anwar N., Ahmad N. (2017). Proliposomes as Pharmaceutical Drug Delivery System: A Brief Review. J. Nanomed. Nanotechnol..

[31] Khan I., Yousaf S., Subramanian S., Korale O., Alhnan M.A., Ahmed W., Taylor K.M.G., Elhissi A. (2015). Proliposome Powders Prepared Using a Slurry Method for the Generation of Beclometasone Dipropionate Liposomes. Int. J. Pharm..

[32] Khan I., Lau K., Bnyan R., Houacine C., Roberts M., Isreb A., Elhissi A., Yousaf S. (2020). A Facile and Novel Approach to Manufacture Paclitaxel-Loaded Proliposome Tablet Formulations of Micro or Nano Vesicles for Nebulization. Pharm. Res..

[33] Byeon J.C., Lee S., Kim T., Ahn J.B., Kim D., Choi J., Park J.-S. (2019). Design of Novel Proliposome Formulation for Antioxidant Peptide, Glutathione with Enhanced Oral Bioavailability and Stability. Drug Deliv..

[34] Karn P.R., Jin S., Lee B.J., Sun B.K., Kim M., Sung J., Hwang S.-J. (2014). Preparation and Evaluation of Cyclosporin A-Containing Proliposomes: A Comparison of the Supercritical Antisolvent Process with the Conventional Film Method. Int. J. Nanomed..

[35] Sheshala R., Hong G.C., Yee W.P., Meka V.S., Thakur R.R.S. (2019). In Situ Forming Phase-Inversion Implants for Sustained Ocular Delivery of Triamcinolone Acetonide. Drug Deliv. Transl. Res..

[36] Abdel-Salam F.S., Elkheshen S.A., Mahmoud A.A., Basalious E.B., Amer M.S., Mostafa A.A., Elkasabgy N.A. (2020). In-Situ Forming Chitosan Implant-Loaded with Raloxifene Hydrochloride and Bioactive Glass Nanoparticles for Treatment of Bone Injuries: Formulation and Biological Evaluation in Animal Model. Int. J. Pharm..

[37] Nekkanti V., Rueda J., Wang Z., Betageri G.V. (2016). Design, Characterization, and In Vivo Pharmacokinetics of Tacrolimus Proliposomes. AAPS PharmSciTech.

[38] Ghoke S.S., Sood R., Kumar N., Pateriya A.K., Bhatia S., Mishra A., Dixit R., Singh V.K., Desai D.N., Kulkarni D.D. (2018). Evaluation of Antiviral Activity of *Ocimum sanctum* and *Acacia arabica* Leaves Extracts Against H9N2 Virus Using Embryonated Chicken Egg Model. BMC Complement. Altern. Med..

[39] Azeem S., Ashraf M., Rasheed M.A., Anjum A.A., Hameed R. (2015). Evaluation of Cytotoxicity and Antiviral Activity of Ivermectin Against Newcastle Disease Virus. Pak. J. Pharm. Sci..

[40] Callison S.A., Hilt D.A., Boynton T.O., Sample B.F., Robison R., Swayne D.E., Jackwood M.W. (2006). Development and Evaluation of a Real-Time Taqman RT-PCR Assay for the Detection of Infectious Bronchitis Virus from Infected Chickens. J. Virol. Methods.

[41] Hani U., Osmani R.A.M., Alqahtani A., Ghazwani M., Rahamathulla M., Almordy S.A., Alsaleh H.A. (2021). 2^3^ Full Factorial Design for Formulation and Evaluation of Floating Oral In Situ Gelling System of Piroxicam. J. Pharm. Innov..

[42] Alyami M.H., Alyami H.S., Abdo A.M., Sabry S.A., El-Nahas H.M., Ayoub M.M. (2024). Maximizing the Use of Ivermectin Transethosomal Cream in the Treatment of Scabies. Pharmaceutics.

[43] Albash R., Abdelbary A.A., Refai H., El-Nabarawi M.A. (2019). Use of Transethosomes for Enhancing the Transdermal Delivery of Olmesartan Medoxomil: In Vitro, Ex Vivo, and In Vivo Evaluation. Int. J. Nanomed..

[44] Marques S.M., Shirodkar R.K., Kumar L. (2023). Analytical ‘Quality-by-Design’ Paradigm in Development of a RP-HPLC Method for the Estimation of Cilnidipine in Nanoformulations: Forced Degradation Studies and Mathematical Modelling of In-Vitro Release Studies. Microchem. J..

[45] Eldin N.E., Elnahas H.M., Mahdy M.A.E., Ishida T. (2015). Liposomal Pemetrexed: Formulation, Characterization and in Vitro Cytotoxicity Studies for Effective Management of Malignant Pleural Mesothelioma. Biol. Pharm. Bull..

[46] Hussein N.R. (2019). Spray-Dried Liposomes: A Study of the Effect of Carbohydrate Carrier and Concentrations on Liposome Size and Drug Entrapment. Zanco J. Med. Sci..

[47] Najlah M., Jain M., Wan K.W., Ahmed W., Albed Alhnan M., Phoenix D.A., Taylor K.M.G., Elhissi A. (2018). Ethanol-Based Proliposome Delivery Systems of Paclitaxel for In Vitro Application Against Brain Cancer Cells. J. Liposome Res..

[48] Nekkanti V., Wang Z., Betageri G.V. (2016). Pharmacokinetic Evaluation of Improved Oral Bioavailability of Valsartan: Proliposomes Versus Self-Nanoemulsifying Drug Delivery System. AAPS PharmSciTech.

[49] Ferrara F., Benedusi M., Cervellati F., Sguizzato M., Montesi L., Bondi A., Drechsler M., Pula W., Valacchi G., Esposito E. (2022). Dimethyl Fumarate-Loaded Transethosomes: A Formulative Study and Preliminary Ex Vivo and In Vivo Evaluation. Int. J. Mol. Sci..

[50] Choudhury D., Tanner M.G., McAughtrie S., Yu F., Mills B., Choudhary T.R., Seth S., Craven T.H., Stone J.M., Mati I.K. (2016). Endoscopic Sensing of Alveolar PH. Biomed. Opt. Express.

[51] Janga K.Y., Jukanti R., Velpula A., Sunkavalli S., Bandari S., Kandadi P., Veerareddy P.R. (2012). Bioavailability Enhancement of Zaleplon via Proliposomes: Role of Surface Charge. Eur. J. Pharm. Biopharm..

[52] Olejnik A., Kapuscinska A., Schroeder G., Nowak I. (2017). Physico–Chemical Characterization of Formulations Containing Endomorphin—2 Derivatives. Amino Acids.

[53] Potluri P., Betageri G.V. (2006). Mixed-Micellar Proliposomal Systems for Enhanced Oral Delivery of Progesterone. Drug Deliv..

[54] Rolim L.A., dos Santos F.C.M., Chaves L.L., Gonçalves M.L.C.M., Freitas-Neto J.L., da Silva do Nascimento A.L., Soares-Sobrinho J.L., de Albuquerque M.M., do Carmo Alves de Lima M., Rolim-Neto P.J. (2015). Preformulation Study of Ivermectin Raw Material. J. Therm. Anal. Calorim..

[55] Santonocito D., Puglia C., Torrisi C., Giuffrida A., Greco V., Castelli F., Sarpietro M.G. (2021). Calorimetric Evaluation of Glycyrrhetic Acid (Ga)- and Stearyl Glycyrrhetinate (Sg)-Loaded Solid Lipid Nanoparticle Interactions with a Model Biomembrane. Molecules.

[56] Haghiralsadat F., Amoabediny G., Sheikhha M.H., Zandieh-Doulabi B., Naderinezhad S., Helder M.N., Forouzanfar T. (2017). New Liposomal Doxorubicin Nanoformulation for Osteosarcoma: Drug Release Kinetic Study Based on Thermo and PH Sensitivity. Chem. Biol. Drug Des..

