# Supplementary material for: Experimental Investigation into the Design, Optimization, Toxicity, and Anti-Viral Efficacy of Proliposomes Loaded with Ivermectin Against Infectious Bronchitis Virus Using an Embryonated Chicken Egg Model
Source: Pharmaceutics. 2025 Jan 25;17(2):165. doi: 10.3390/pharmaceutics17020165 (PMC11859826; doi:10.3390/pharmaceutics17020165)

Figure S1. Linear correlation plots between actual and predicted values for **(a)** Y_1 response, (**b**)Y_2 response, **(c)** Y_3 response, **(d)** Y_4 response, **(e)** Y_5 response, **(f)** Y_6 response.

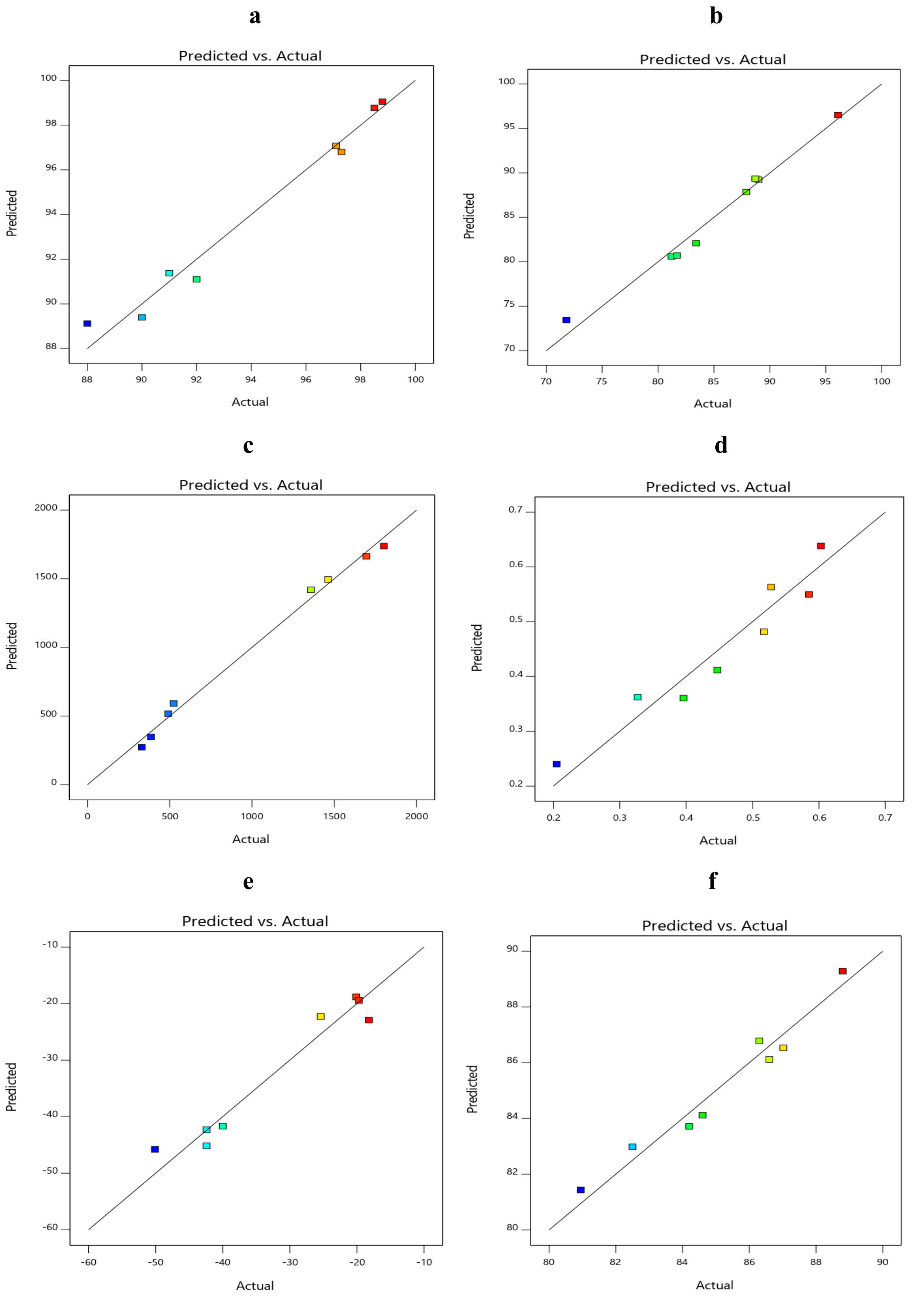

Figure S2. PS and ZP of IVM-loaded PLs formulations. **(a)** PS of SPC based IVM-Loaded PLs, **(b)** PS of DPPC based IVM-Loaded PLs, **(c)** ZP of SPC based IVM-Loaded PLs, **(d)** ZP of DPPC based IVM-Loaded PLs.

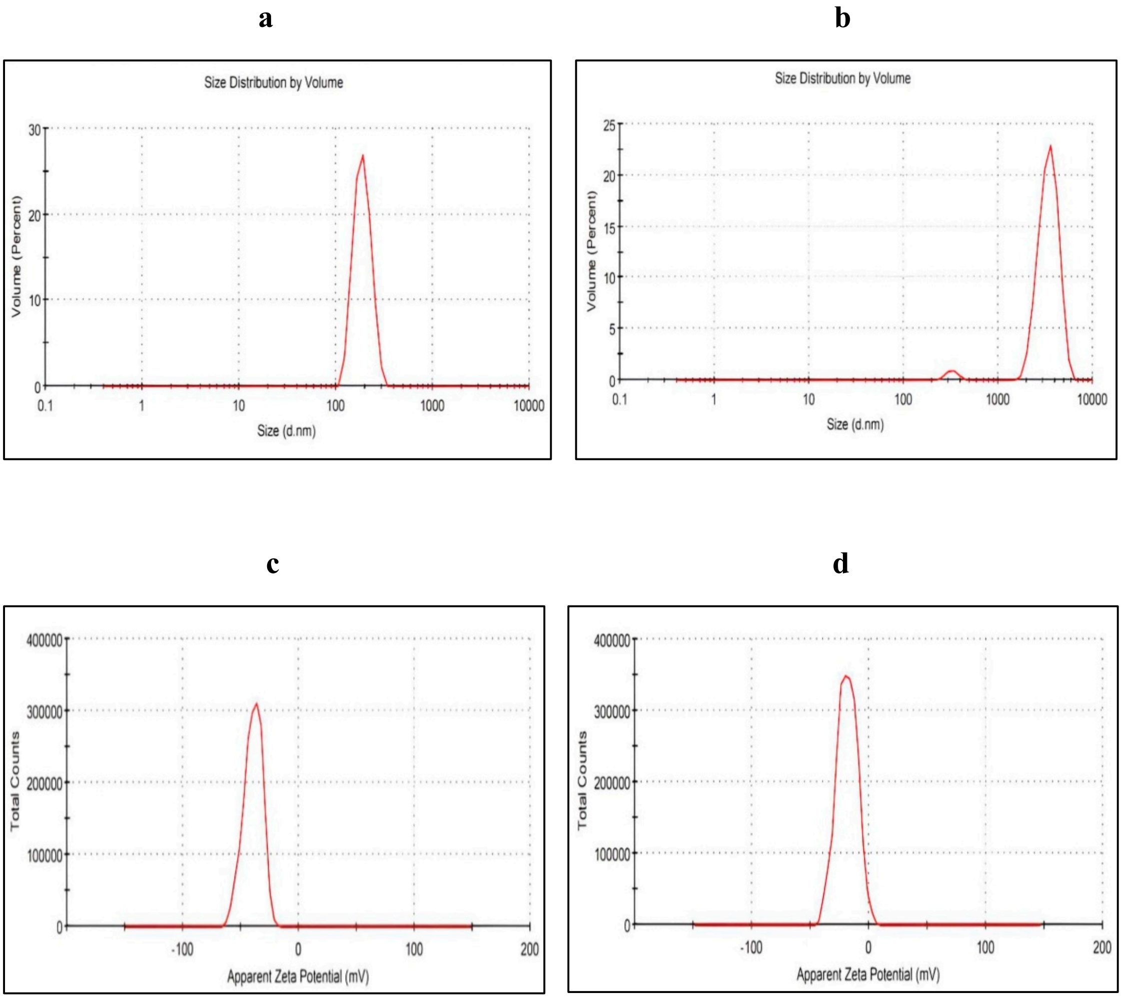

Figure S3. In-vitro drug release profiles of IVM-loaded PLs formulations (n=3±SD).

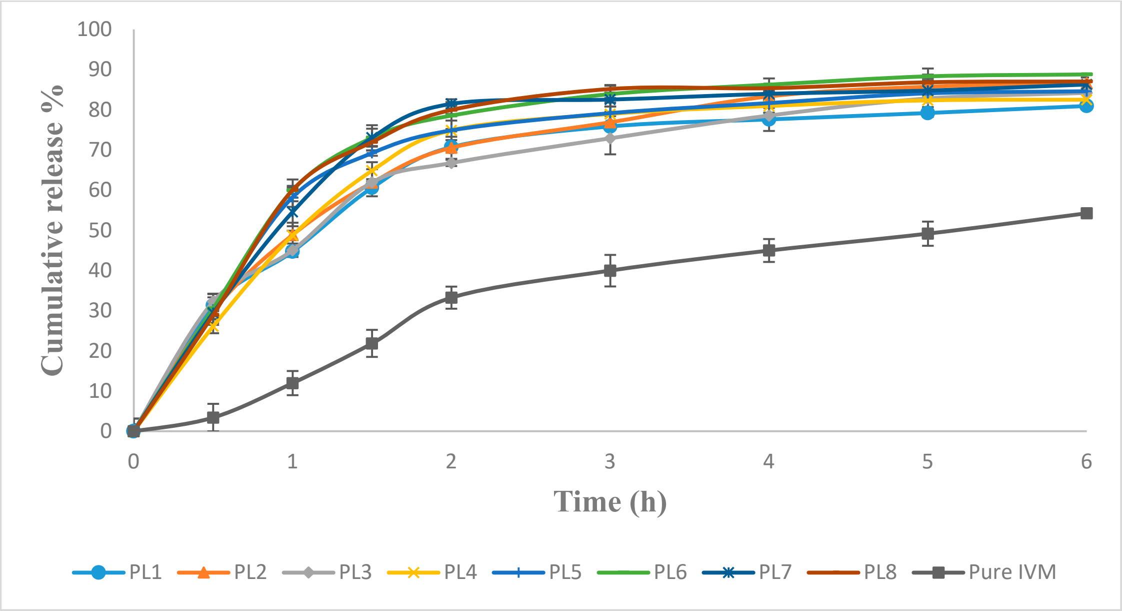

Supplement: Supplementary file 1 [file pharmaceutics-17-00165-s001.zip › pharmaceutics-3357462-supplementary.pdf]
